# High intensity, circuit-type integrated neuromuscular training alters energy balance and reduces body mass and fat in obese women: A 10-month training-detraining randomized controlled trial

**DOI:** 10.1371/journal.pone.0202390

**Published:** 2018-08-23

**Authors:** Alexios Batrakoulis, Athanasios Z. Jamurtas, Kalliopi Georgakouli, Dimitrios Draganidis, Chariklia K. Deli, Konstantinos Papanikolaou, Alexandra Avloniti, Athanasios Chatzinikolaou, Diamanda Leontsini, Panagiotis Tsimeas, Nikolaos Comoutos, Vassilios Bouglas, Maria Michalopoulou, Ioannis G. Fatouros

**Affiliations:** 1 School of Physical Education and Sport Sciences, University of Thessaly, Karies, Trikala, Greece; 2 School of Physical Education and Sport Sciences, Democritus University of Thrace, Komotini, Greece; TNO, NETHERLANDS

## Abstract

This randomized controlled trial examined body mass, body composition, energy balance and performance responses of previously sedentary overweight/obese women to a circuit-type integrated neuromuscular training program with alternative modalities. Forty-nine healthy overweight or class I obese females (36.4±4.4 yrs) were randomly assigned to either a control (N = 21), training (N = 14) or training-detraining (N = 14) group. In weeks 1–20, the training groups trained three times/week using 10–12 whole-body exercises of progressively increased intensity/volume, organized in timed interval circuit form. In weeks 21–40, the training group continued training whereas the training-detraining group not. Heart rate, perceived exertion, blood lactate, exertion, oxygen consumption and excess post-exercise oxygen consumption were measured for one session/phase/person and exercise energy expenditure was calculated. Energy intake, habitual physical activity, resting metabolic rate, body composition, body mass, strength and maximal oxygen consumption were measured at baseline, mid-intervention and post-intervention. A two-way repeated measures ANOVA was used to determine differences between three time points and three groups. In C, VO_2max_ declined (p<0.013) and body fat (p<0.008), waist (p<0.059) and hip (p<0.012) circumferences increased after 40 weeks compared to baseline. Training reduced body mass (6%, p<0.001), body fat (~5.5%, p<0.001) and increased fat-free mass (1.2–3.4%, p<0.05), strength (27.2%, p<0.001) and endurance (26.8%, p<0.001) after a 10-month implementation period using a metabolic overload of only 5–12 metabolic equivalents of task-hours per week. Training induced a long-term negative energy balance during an exercise and a non-exercise day due to an elevation of resting metabolic rate (6%-10%, p<0.05) and exercise-related energy expenditure. Training had an 8% and 94% attrition and attendance rates, respectively. Training-induced gains were attenuated but not lost following a 5-month detraining. A 10-month implementation of a high-intensity interval type training program elicited both endurance and musculoskeletal gains and resulted in a long-term negative energy balance that induced a progressive and sustained reduction of body and fat mass.

**Trial Registration**: ClinicalTrials.gov NCT03134781

## Introduction

Obesity epidemic, a major health issue predisposing to cardiovascular diseases, type 2 diabetes and other pathologies [1], has doubled worldwide over the last decade and thus represents a serious threat for the survival of public health care systems [2]. Developing effective strategies to prevent and/or manage obesity is important. Obesity represents an imbalance between energy intake and expenditure, in favour of the former, over a given period of time [3]. Anti-obesity interventions should strive for a 5–10% reduction in body mass [4] by promoting lifestyle changes favouring energy expenditure over feeding [5]. Although a decline of this magnitude may not normalize body mass of an obese adult, it will improve risk factors associated with obesity-related diseases [6]. Almost 50% of Caucasian women in developed countries are classified as overweight, inactive, and demonstrate increased likelihood to become obese rendering them ideal candidates to develop cardiovascular and metabolic disorders [7–10].

Exercise interventions have mainly used systematic physical activity and/or continuous endurance exercise of moderate-to-high intensity that gradually increases to ≥250 min/week may improve body composition, promote weight loss, prevent weight regain and reduce risk factors for obesity-related disorders even without a weight loss [6]. This type of exercise training induces significant weight loss only when applied systematically at a high weekly volume (>13 MET-hours/week) [11–13]. The inclusion of resistance exercise training to such programs may further increase performance, skeletal muscle mass, resting metabolic rate and energy expenditure and thus improve body composition and overall health [14]. Resistance exercise protocols incorporating whole-body movements that target the activation of the entire neuromuscular system may also improve the functional capacity to perform activities of daily living of people demonstrating neuromuscular limitations and reduced mobility such as the obese [15,16]. However, when continuous endurance and resistance exercise training programs are applied as discrete exercise modes within the same training regimen are time-consuming and may have high attrition and low compliance rates [17]. On the other hand, high-intensity exercise protocols (HIIT) incorporating mainly cardiovascular activities performed in interval and/or circuit fashion have been proposed [18–20] because they are time-efficient [21] and they also improve aerobic capacity [22], body composition [23], resting metabolic rate[24] and skeletal muscle mitochondrial metabolism [25] in healthy, sedentary overweight and obese adults. HIIT-induced changes in mitochondrial function may explain its greater effectiveness in inducing positive metabolic adaptations compared to traditional endurance and/or resistance exercise training protocols even when no diet intervention is applied in weight loss trials [26].

The present study applied a high-intensity exercise protocol performed in interval fashion that utilized integrated neuromuscular resistance exercises with whole-body movements, i.e. a high-intensity circuit-type neuromuscular exercise training protocol (CINT) of limited duration to combine the metabolic and performance adaptations of HIIT and resistance exercise in a time-efficient manner. CINT incorporated primal full-body movements using alternative modes and nontraditional implements [27] performed in a progressive manner and using a small-group setting. We hypothesized that CINT may be effective to create an energy deficit and promote health and overall well-being with a limited attrition rate due to its interval nature and reduced weekly volume in otherwise healthy overweight/obese Caucasian women. Therefore, the objective of this investigation was to determine the effects of a CINT protocol with whole-body exercises using alternative modalities [28] on (i) body mass, (ii) body composition, (iii) resting metabolic rate, (iv) overall energy balance and (v) performance of previously inactive, overweight/obese women.

## Materials and methods

### Ethics statement

Participants signed a consent form after they were informed of all risks, discomforts and benefits involved in the study. Procedures were in agreement with the 1975 Declaration of Helsinki, as revised in 2000, and approval was granted by the Institutional Ethics Committee of the Department of Physical Education and Sports Sciences of the University of Thessaly (protocol ID 1025/15-7-2015).

### Participants and research design

**Fig 1:**
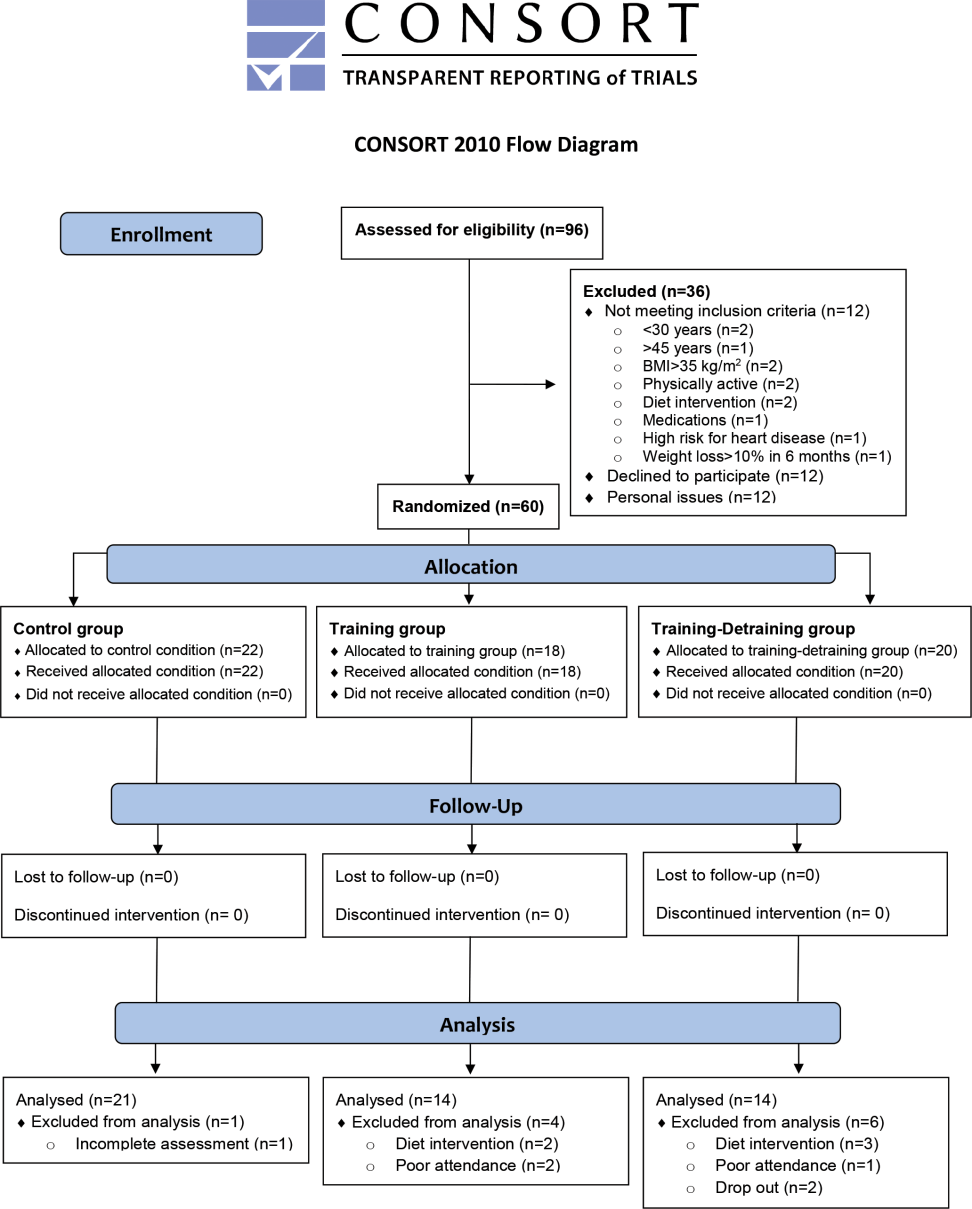
CONSORT diagram of the study.

**Fig 2:**
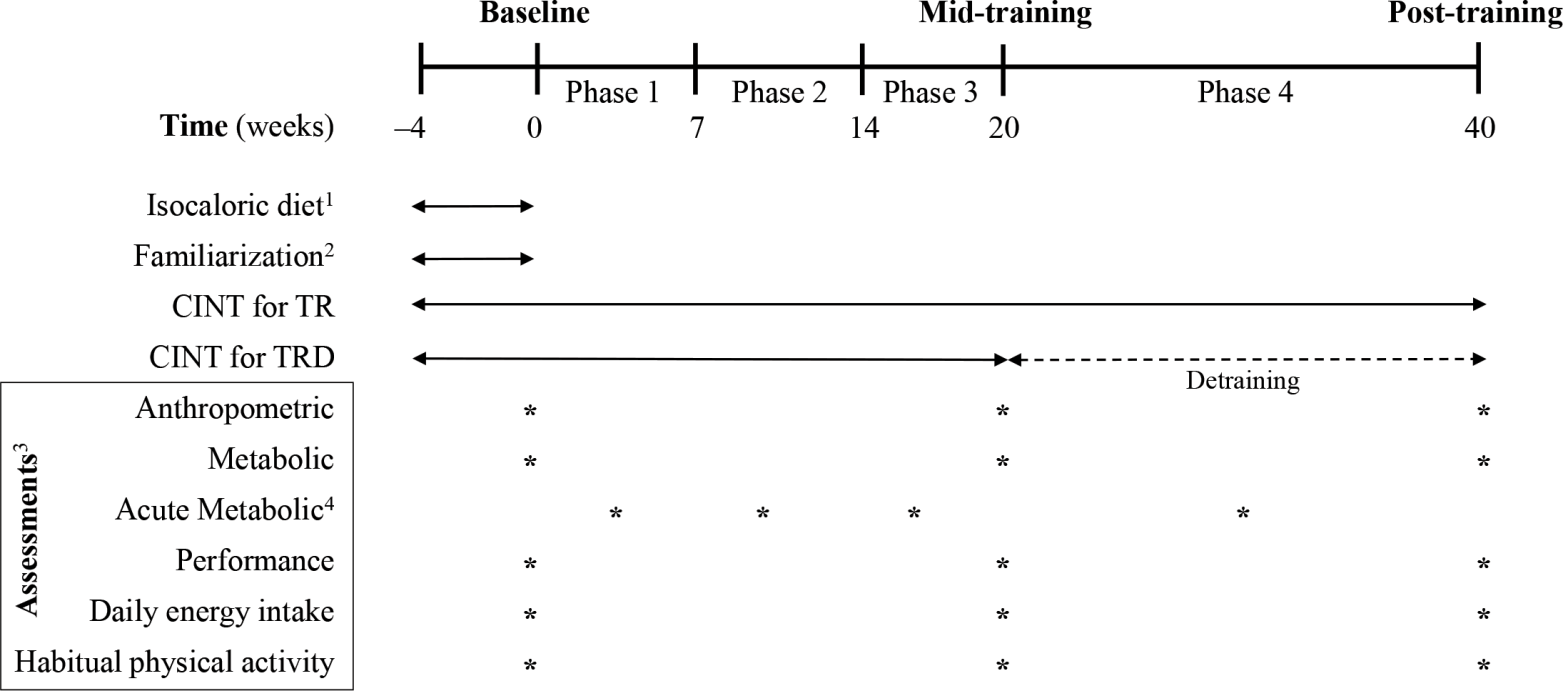
Experimental flowchart. C, control group; TR, training group (40 weeks); TRD, training (20 weeks)—detraining (20 weeks) group; CINT, circuit integrated neuromuscular training; RMR, resting metabolic rate; ^1^for all groups (4-week adaptation period); ^2^only for TR and TRD; ^3^for all groups; ^4^acute metabolic measurements for TR and TRD (1 session per participant in each training phase: perceived exertion and heart rate measurement during the exercise session, oxygen consumption and blood lactate concentration before, during and after a session and excess post-exercise oxygen consumption after a session).

Fig 1 shows the CONSORT diagram of the study and Fig 2 illustrates the experimental flowchart of the study. The main goal of this investigation was to evaluate the efficacy of a circuit integrated neuromuscular training protocol with whole-body exercises using alternative modalities on energy balance of obese women and not to compare it with other exercise approaches used for weight management. A controlled, randomized, three-group, repeated-measures design was employed at the facilities of the University of Thessaly (recruitment period: July/August 2015) and the follow-up period was from February 2016 to July 2016. The study (DoIT trial) was registered in ClinicalTrials.gov [URL: https://www.clinicaltrials.gov/ct2/show/NCT03134781?term=NCT03134781&rank=1] (ID: NCT03134781). Due to misinterpretation of the relevant policy the registration of this study as a clinical trial was delayed. The authors confirm that all ongoing and related trials for this intervention are registered.

Based on previous training studies with overweight individuals (e.g., [15,20,22,23]), a preliminary power analysis (effect size >0.55, probability error of 0.05, two-tailed alpha level, power of 0.9) using the G*Power 3.0.10 program for three groups and three measurements points suggested that a sample of 36–40 participants was necessary to identify statistically meaningful trial effects. Participants were recruited using fliers (posted in the local community), social media and by word of mouth. Women participated in the study if they: (a) were inactive (<7,500 steps·day^-1^; VO_2max_ <30 ml·kg^-1^·min^-1^; accelerometry-based moderate-to-vigorous physical activity <30 min·day^-1^), b) were healthy, premenopausal and aged 30–45 years, c) were overweight or obese class 1 [Body Mass Index (BMI) 25.1–34.9 kg/m^2^], d) had medical clearance for strenuous physical training, e) were non-smokers for ≥6 months before the study, f) were not following a diet intervention or using nutritional supplements/medications before (≥6 months) and during the study, g) had no weight loss greater >10% of body mass before (≤6 months) the study and h) had no symptoms of depression. Participants were excluded from the study if: a) participated in ≤80% of total exercise sessions, and b) adhered to a nutritional intervention during the study. Ninety-six females were interviewed, 60 were recruited (12 were not interested in participating, 12 did not meet the inclusion/exclusion criteria and 12 were excluded due to personal issues) and 49 completed it [data from 11 women were not used because of altered energy intake during the study (5 women), poor attendance (3 women), drop out (2 women), and failure to participate in all measurements (1 woman)] (Fig 2). Table 1 shows participants’ baseline characteristics.

**Table 1:** Participants’ baseline characteristics.

	C (N = 21)	TR (N = 14)	TRD (N = 14)
Age (years)	36.0 ± 4.2	36.4 ± 5.0	36.9 ± 4.3
Body mass (kg)	80.2 ± 8.9	78.0 ± 9.9	78.7 ± 7.9
Body height (m)	1.65 ± 0.5	1.66 ± 0.5	1.64 ± 0.6
BMI (kg·m^-2^)	29.6 ± 3.0	28.2 ± 2.8	29.1 ± 3.0
PA (steps·day^-1^)	6,399.7 ± 1,851.3	6,330.7 ± 1,041.5	6,870.0 ± 2,030.6
Body fat (%)	46.7 ± 6.5	47.5 ± 3.2	46.2 ± 3.9
VO_2max_ (ml·kg^-1^·min^-1^)	26.1 ± 3.2	26.1 ± 4.4	27.4 ± 3.2
1RM Leg press (kg)	133.8 ± 29.7	124.6 ± 22.4	131.4 ± 18.5

C, control group; TR, trained group; TRD, trained-detrained group; BMI, body mass index; PA, physical activity; VO_2max_, maximal oxygen uptake; 1RM, one repetition maximum.

Participants were randomly assigned to either (i) a control group (C, N = 21; participated only in measurements), (ii) a training group (TR, N = 14), or (iii) a training-detraining group (TRD, N = 14). Randomization was achieved by a random-numbers table (allocation sequence conducted by an independent researcher and was concealed until interventions were assigned). The first author (AB) enrolled and assigned participants to interventions. Initially, participants followed an isocaloric diet adaptation period and they were familiarized with the CINT protocol (4 weeks). During the first 20 weeks, TR and TRD followed the CINT exercise protocol. In weeks 21–40, TR continued the CINT protocol whereas TRD abstained from training (detraining). Anthropometric, metabolic, performance, daily energy intake, and habitual physical activity assessments were performed at baseline and at 20 and 40 weeks of intervention (five days after the last training session). Blood lactate concentration, exercise oxygen consumption (VO_2_), heart rate and ratings of perceived exertion were measured before, during and after a training session in each training phase (one session/person/phase). Excess post-exercise oxygen consumption was assessed after a training session in each training phase (one session/person/phase).

### Exercise intervention

Tables 2 and 3 illustrate the configuration of the training protocol across all phases. A supervised, small-group (5–10 women/session) training protocol was performed three times/week (with a 48-hour recovery between sessions), with the use of asynchronous music, for 40 weeks. Exercises incorporated fundamental movement patterns using bodyweight as resistance [19,20] or adjunct portable modalities [28]. Each session was preceded by a 10-min warm-up (low-intensity endurance exercise, stretching exercises and mobility exercises) and followed by a 5-min cool-down period (walking/stretching exercises). Exercises (~10-12/session) were performed in circuit fashion using prescribed time (20–40 sec) of effort and recovery intervals. Participants performed as many repetitions as possible at each station with proper form at a controlled, moderate speed. Each session consisted of alternate stations emphasizing cardiovascular, resistance and neuromotor exercises. For resistance-type multi-joint exercises, participants were encouraged to use a comfortable resistance at the beginning of the study and progressed to heavier loads that allowed them to complete the desired exercise duration at each station.

**Table 2:** The characteristics of the training protocol across all training phases.

Training Parameters	Phase 1 (Week 1–7)	Phase 2 (Week 8–14)	Phase 3 (Week 15–20)	Phase 4 (Week 21–40)
Session duration (min)	23.0	38.0	41.0	41.0
Effort time (min)^a^	6.66	16.5	24.0	24.0
Recovery time (min)^b^	16.34	21.5	17.0	17.0
Work-to-rest ratio	1:2	1:1	2:1	2:1
Work interval (sec)	20.0	30.0	40.0	40.0
Rest interval (sec)	40.0	30.0	20.0	20.0
Exercises amount	10	11	12	12
Rounds	2	3	3	3
Rest time/round (min)	3.0	2.5	2.5	2.0
Movement number^c^	Maximal	Maximal	Maximal	Maximal

^a^Effort time = session duration–recovery time.

^b^Recovery time = session duration–effort time.

^c^Maximal number of movements during efforts time.

**Table 3:** The exercises of the training protocol across all training phases.

	Exercises			
Adjunct Modalities	Phase 1	Phase 2	Phase 3	Phase 4
1. Balance Ball	Over dome ankle touch	Straddle jump	Split jack	Over dome hand touch
2. Suspension Exercise Device	Neutral grip row	Wide grip row	Y deltoid raise	Chest press
3. Kettlebell	Sumo deadlift	Sumo deadlift high pull	Two-arm swing	Two-arm snatch
4. Bodyweight	Straight-arm plank	Forearm plank	Straight-arm reverse plank	Side plank rotation
5. Speed Ladder	Low knee skip	Lateral shuffle	Heel flick	High knee skip
6. Battling Rope	Bilateral wave	Alternating wave	Side-to-side wave	Slam
7. Medicine Ball	Alternating static lunge	Forward lunge with press	Lunge to chest pass	Twisting chop
8. Foam Roller	Forearm plank	Forearm plank with leg lift	Shifting Plank	Forearm plank with leg lift
9. Bodyweight	Jumping jack	Split jack	Ice skater	Burpee
10. Resistance Band with Stick	Squat to overhead press	Lateral shuffle press	Hockey slap shot	Axe chop
11. Resistance Band		Squat row	Reverse fly with lunge	Squat to overhead press
12. Medicine Ball			Squat throw	Swing

During the first 20 weeks, training was divided in three phases characterized by a progressive increase in exercise intensity and volume. During the second 20 weeks (phase 4), training maintained the intensity and volume of phase 3 but the work-to-rest ratio was varied bi-weekly. Heart rate was monitored (Polar Team Solution, Polar Electro-Oy, Kempele, Finland) throughout each session and mean and maximal heart rates were recorded. Verbal encouragement was given by the investigators and participants were guided to keep an intensity ≥65% of heart rate reserve throughout the session. Rates of perceived exertion were recorded for each round and mean exertion was calculated. Exercise intensity was calculated as mean heart rate (percentage of maximal heart rate obtained during VO_2max_ testing), percentage (%) of heart rate reserve, RPE, % of VO_2peak_, metabolic equivalents of task (METs), and blood lactate accumulation for all participants.

### Measurements

Height and body mass were measured to the nearest 0.1 cm and 0.1 kg, respectively, using a beam scale (SECA 220, Hamburg, Germany) and BMI was calculated. Waist and hip circumferences were measured using a Gullick II tape and the waist-to-hip ratio was calculated. The fat and fat-free mass were measured using the 12.2 enCORE software package of a whole-body dual-energy X-ray absorptiometry scanner (Lunar Prodigy Advance, GE Lunar Healthcare Corp., Madison, WI, USA) as described [29].

Strength (one repetition maximal, 1RM) was measured bilaterally on a horizontal leg press machine (Panatta Sport, Apiro, Italy) using standard procedures [14] with an intra-class correlation coefficient for test-retest trials of 0.88. VO_2peak_ was assessed during a graded maximal exercise testing using the modified Balke protocol [30] on a treadmill (Precor 954i, Woodinville, WA, USA). During testing, expired O_2_ and CO_2_ concentrations were analyzed by a portable open-circuit spirometry system (Fitmate Pro, Cosmed, Chicago, IL, USA) in 15-second intervals and heart rate, blood pressure and rates of perceived exertion were recorded. Attainment of VO_2peak_ was verified if standard criteria were met [31]. For resting metabolic rate assessment, resting VO_2_/CO_2_ were measured in the morning (07.00–09.00) after an overnight fast using an open-circuit indirect calorimeter with a ventilated hood system (Fitmate Pro, Cosmed, Chicago, IL, USA) as described [32] and the 24-hour resting metabolic rate was calculated using the Weir equation [33].

Exercise energy cost and excess post-exercise oxygen consumption was measured using portable indirect calorimetry (Vmax_ST_, Sensormedics, Yorba Linda, CA) as described [32]. Total energy expenditure of an exercise session was estimated by summing a) the aerobic energy expenditure during exercise which was estimated using a constant value of 21.14 kJ (5.05 kcal/)/liter oxygen [34], b) the anaerobic energy expenditure using resting and post-exercise blood lactate concentration measurements [35], and c) excess post-exercise oxygen consumption. For lactate measurement, blood samples were collected pre-, mid- and post-exercise session (3 min post-exercise). Investigators collected the blood sample after they had thoroughly cleaned, disinfected and dried subjects’ whole hands and the single finger (to avoid any potential contamination due to interference with sweat) used for blood sampling. Thereafter, a lancet was used to puncture participants’ skin at the finger, the first blood drop was directed on the measurement strip and the blood lactate concentration was analyzed using a hand-portable analyzer (Accutrend Plus, Roche Diagnostics, Switzerland) within a few seconds following collection.

Seven-day habitual physical activity level was determined via accelerometry (GT3X-BT, ActiGraph, FL, USA) as described [36]. Accelerometers were placed into adjustable belts and were over the right hip during the measurement (throughout the day except during bathing and sleeping). Participants’ data were included in the analysis, if they had ≥4 days and ≥10 wear hours/day [37]. Non-wear time was estimated using algorithms for vector magnitude data as described [38]. Daily activity and sedentary time were calculated based on four vector magnitude data and expressed as steps/day and time in sedentary (<199 cpm), light (200–2,689 cpm), moderate (2,690–6,166 cpm), vigorous (6,167–9,642 cpm) and moderate-to-vigorous (≥2,690 cpm) physical activity [39]. The ActiLife 6 software was used to start the accelerometers and access data (60-s epoch length). Participants were encouraged to maintain their usual daily physical activity throughout the study.

All participants were asked to follow an isocaloric diet (based on resting metabolic rate measurements and habitual physical activity). A dietitian provided instructions on how to adapt to a weight maintenance diet (55–60% carbohydrate, 15–20% protein, 20%-25% fat), in the form of nutritional equivalents during an initial adaptation period when body mass was monitored to verify the accuracy of the assigned energy approach (it was re-adjusted at 20 weeks). To measure caloric intake, participants submitted 7-day diet recalls following training on how to record food/fluid consumption by a registered dietitian. Participants were instructed to maintain the same feeding pattern and to avoid any dietary interventions during the entire study. Diet recalls were analysed for energy and macronutrient (carbohydrate, fat, and protein) intake using the nutritional analysis software “Science Fit Diet 200A” (Science Technologies, Athens, Greece).

Energy balance during an exercise and a non-exercise day was estimated as calories consumed through daily diet minus calories expended as resting metabolic rate, exercise energy cost and habitual physical activity.

### Statistical analysis

Data were analyzed using SPSS 22.0. Assumptions of sphericity were tested using Mauchley’s test and if violated degrees of freedom were corrected using Greenhouse-Geisser estimates of sphericity. A two-way (treatment×time) repeated measures ANOVA was used to determine differences between three time points (baseline, 20 weeks, 40 weeks) and three groups (control, training, training-detraining). When a significant interaction was detected, data were subsequently analyzed using a Bonferroni post-hoc test. Significance was accepted at *p*≤0.05. Analysis of Covariance (ANCOVA, all time points in the three groups) was used to adjust body mass by daily caloric consumption and daily physical activity. Effect sizes (ES) and confidence intervals (CI) were calculated for all dependent variables using the Hedge’s g method corrected for bias. ES was interpreted as none, small, medium-sized and large for values 0.00–0.19, 0.20–0.49, 0.50–0.79 and ≥0.8, respectively. Data are presented as means±standard deviation.

## Results

A 94% compliance and an 8% drop-out rate were recorded for the training protocol during implementation. No differences were detected among groups for all dependent variables at baseline and as such analysis of covariance was not necessary. No injuries or other exercise-induced health problems were recorded.

Table 4 presents the overload, physiological, and metabolic characteristics of the exercise protocol across all training phases. The mean training heart rate was similar in TR and TRD throughout training. Mean training heart rate increased progressively from phase 1 through phase 3 and stabilized thereafter in TR. Blood lactate concentration during a training session exhibited a progressive rise in all phases. Blood lactate concentration measured at mid- and post-exercise was of lower magnitude in phase 1 compared to the other phases but there were no differences between phases 2, 3 and 4. Mean minute ventilation, VO_2_, RER, METs, rates of perceived exertion, total energy expenditure during an exercise session increased progressively from phase 1 to 4.

**Table 4:** Overload, physiological, and metabolic characteristics of the training protocol across all training phases.

Variables	Phase 1 (Week 1–7)	Phase 2 (Week 8–14)	Phase 3 (Week 15–20)	Phase 4 (Week 21–40)	Comparison between phases
Mean HR (beats·min^-1^)	118.5 ± 9.2	130.8 ± 12.6^a^	143.2 ± 12.1^b^^,^^d^	151.1 ± 11.3^c^^,^^e^	Phases 1 vs 2: p<0.05; CI: -1.94, -0.34; ES = -1.14 Phases 1 vs 3: p<0.001; CI: -2.80, -1.01; ES = -1.91 Phases 1 vs 4: p<0.001; CI: -3.99, -1.86; ES = -2.93 Phases 2 vs 3: p = 0.011; CI: -1.73, -0.16; ES = -0.94 Phases 2 vs 4: p = 0.001; CI: -2.67, -0.91; ES = -1.79
Mean HR as % maxHR (as % HR reserve)	72.5 ± 7.9 (57.9 ± 7.4)	79.7 ± 6.4 (69.1 ± 5.9)	87.0 ± 6.3^b^^,^^d^ (79.1 ± 4.3)	87.5 ± 4.7^c^^,^^e^ (79.5± 4.7)	Phases 1 vs 3: p<0.001; CI: -2.89, -1.08; ES = -1.98 Phases 1 vs 4: p = 0.001; CI: -2.88, -1.07; ES = -1.98 Phases 2 vs 3: p = 0.01; CI: -2.42, -0.73; ES = -1.58 Phases 2 vs 4: p = 0.013; CI: -2.39, -0.70; ES = -1.54
maxHR (beats·min^-1^)	150.7 ± 12.2	157.6 ± 11.8	164.9 ± 11.6^b^^,^^d^	172.5 ± 9.9^c^^,^^e^	Phases 1 vs 3: p = 0.001; CI: -1.76, -0.20; ES = -0.98 Phases 1 vs 4: p = 0.001; CI: -2.48, -0.77; ES = -1.63 Phases 2 vs 3: p = 0.007; CI: -1.33, 0.18; ES = -0.57 Phases 2 vs 4: p = 0.007; CI: -2.11, -0.48; ES = -1.30
Blood lactate concentration (mM)					
Pre-exercise	1.61 ± 0.41	1.54 ± 0.25	1.36 ± 0.33	1.53 ± 0.40	
Mid-exercise	8.02 ± 0.90	11.57 ± 0.72^a^	12.31 ± 1.63^b^	12.42 ± 1.40^c^	Phases 1 vs 2: p<0.001; CI: -5.92, -3.12; ES = -4.52 Phases 1 vs 3: p<0.001; CI: -4.31, -2.08; ES = -3.20 Phases 1 vs 4: p<0.001; CI: -4.65, -2.30; ES = -3.48
Post-exercise	8.99 ± 1.09	11.31 ± 0.77^a^	11.78 ± 1.94^b^	11.99 ± 1.40^c^	Phases 1 vs 2: p<0.001; CI: -3.44, -1.48; ES = -2.46 Phases 1 vs 3: p = 0.001; CI: -2.55, -0.82; ES = -1.69 Phases 1 vs 4: p<0.001; CI: -3.38, -1.43; ES = -2.41
VE (L·min^-1^)	55.71 ± 6.9	62.00 ± 7.0^a^	68.71 ± 5.9^b^^,^^d^	73.50 ± 5.5^c^^,^^e^	Phases 1 vs 2: p<0.001; CI: -1.58, -0.04; ES = -0.81 Phases 1 vs 3: p<0.001; CI: -2.68, -0.92; ES = -1.80 Phases 1 vs 4: p<0.001; CI: -3.66, -1.63; ES = -2.65 Phases 2 vs 3: p<0.001; CI: -1.69, -0.13; ES = -0.91 Phases 2 vs 4: p = 0.001; CI: -2.53, -0.81; ES = -1.67
VO_2_ (mL·kg^-1^·min^-1^)	18.05 ± 1.9	19.26 ± 1.9^a^	23.64 ± 2.^b^^,^^d^	23.84 ± 2.4^c^^,^^e^	Phases 1 vs 2: p<0.001; CI: -1,38, 0,14; ES = -0,62 Phases 1 vs 3: p<0.001; CI: -3,48, -1,50; ES = -2,49 Phases 1 vs 4: p<0.001; CI: -3,66, -1,63; ES = -2,65 Phases 2 vs 3: p<0.001; CI: -2,77, -0,99; ES = -1,88 Phases 2 vs 4: p = 0.001; CI: -2,92, -1,10; ES = -2,01
RER	1.04 ± 0.06	1.08 ± 0.05^a^	1.10 ± 0.06^b^^,^^d^	1.11 ± 0.07^c^	Phases 1 vs 2: p = 0.001; CI: -1.35, 0.16; ES = -0.60 Phases 1 vs 3: p = 0.001; CI: -1.67, -0.12; ES = -0.89 Phases 1 vs 4: p = 0.008; CI: -1.75, -0.19; ES = -0.97 Phases 2 vs 3: p<0.05; CI: -1.07, 0.42; ES = -0.32
METs (MET·hours)	5.16(1.55)±0.6	5.50(2.75)±0.5^a^	6.75(4.05)±0.7^b^^,^^d^	6.78(4.07)±0.7^c^^,^^e^	Phases 1 vs 2: p<0.001; CI: -1.35, 0.16; ES = -0.60 Phases 1 vs 3: p<0.001; CI: -3.53, -1.54; ES = -2.54 Phases 1 vs 4: p<0.001; CI: -3.59, -1.58; ES = -2.59 Phases 2 vs 3: p<0.001; CI: -2.75, -0.97; ES = -1.86 Phases 2 vs 4: p = 0.001; CI: -2.80, -1.01; ES = -1.91
MET·hours·week^-1^	4.65	8.25	12.15	12.21	
RPE	13.71 ± 1.0	14.86 ± 0.9^a^	16.00 ± 0.9^b^^,^^d^	16.14 ± 0.5^c^^,^^e^	Phases 1 vs 2: p<0.001; CI: -1.67, -0.12; ES = -0.89 Phases 1 vs 3: p<0.001; CI: -2.89, -1.08; ES = -1.99 Phases 1 vs 4: p<0.001; CI: -3.37, -1.42; ES = -2.40 Phases 2 vs 3: p<0.001; CI: -1.83, -0.25; ES = -1.04 Phases 2 vs 4: p<0.05; CI: -2.17, -0.53; ES = -2.56
TEE (kcal)	164.9 ± 17.6	326.8 ± 26.9^a^	411.2 ± 45.9^b^^,^^d^	385.1 ± 36.2^c^^,^^e^	Phases 1 vs 2: p<0.001; CI: -6.46, -3.47; ES = -4.96 Phases 1 vs 3: p<0.001; CI: -8.96, -5.01; ES = -6.98 Phases 1 vs 4: p<0.001; CI: -9.58, -5.39; ES = -7.49 Phases 2 vs 3: p<0.001; CI: -3.53, -1.54; ES = -2.53 Phases 2 vs 4: p<0.001; CI: -3.09, -1.22; ES = -2.15
AEE	133.2 ± 17.0	273.7 ± 26.1^a^	348.3 ± 44.3^b^^,^^d^	322.9 ± 34.1^c^^,^^e^	Phases 1 vs 2: p<0.001; CI: -6.84, -3.71; ES = -5.28 Phases 1 vs 3: p<0.001; CI: -8.18, -4.53; ES = -6.35 Phases 1 vs 4: p<0.001; CI: -8.73, -4.87; ES = -6.80 Phases 2 vs 3: p<0.001; CI: -2.68, -0.93; ES = -1.80 Phases 2 vs 4: p = 0.017; CI: -2.15, -0.51; ES = -1.33
ANEE	20.7 ± 3.0	27.0 ± 3.9^a^	28.3 ± 3.3^b^	26.9 ± 3.8^c^	Phases 1 vs 2: p<0.05; CI: -2.58, -0.85; ES = -1.71 Phases 1 vs 3: p = 0.001; CI: -3.34, -1.40; ES = -2.37 Phases 1 vs 4: p = 0.001; CI: -2.68, -0.92; ES = -1.80
EPOC	11.0 ± 2.8	26.1 ± 4.6^a^	34.7 ± 3.4^b^^,^^d^	35.3 ± 3.8^c^^,^^e^	Phases 1 vs 2: p<0.001; CI: -4.23, -2.02; ES = -3.12 Phases 1 vs 3: p<0.001; CI: -8.57, -4.77; ES = -6.67 Phases 1 vs 4: p<0.001; CI: -8.41, -4.68; ES = -6.54 Phases 2 vs 3: p<0.001; CI: -2.64, -0.89; ES = -1.77 Phases 2 vs 4: p = 0.001; CI: -2.73, -0.96; ES = -1.84
EE (kcal/min)	9.2 ± 1.1	10.9 ± 1.5	11.4 ± 1.3	10.7 ± 1.0	

HR, heart rate; maxHR, maximal heart rate; VE, mean minute ventilation; VO_2_, mean oxygen consumption; RER, respiratory exchange ration; METs, metabolic equivalent of task; RPE, rates of perceived exertion; TEE, total energy expenditure; AEE, aerobic energy expenditure; ANEE, anaerobic energy expenditure; EPOC, excess post-exercise oxygen consumption; EE, energy expenditure; CI, confidence intervals; ES, effect size;

^a^denotes a difference between phases 1 and 2 at p<0.05 or at p<0.01;

^b^denotes a difference between phases 1 and 3 at p<0.05 or at p<0.01;

^c^denotes a difference between phases 1 and 4 at p<0.05 or at p<0.01;

^d^denotes a difference between phases 2 and 3 at p<0.05 or at p<0.01;

^e^denotes a difference between phases 2 and 4 at p<0.05 or at p<0.01.

**Fig 3:**
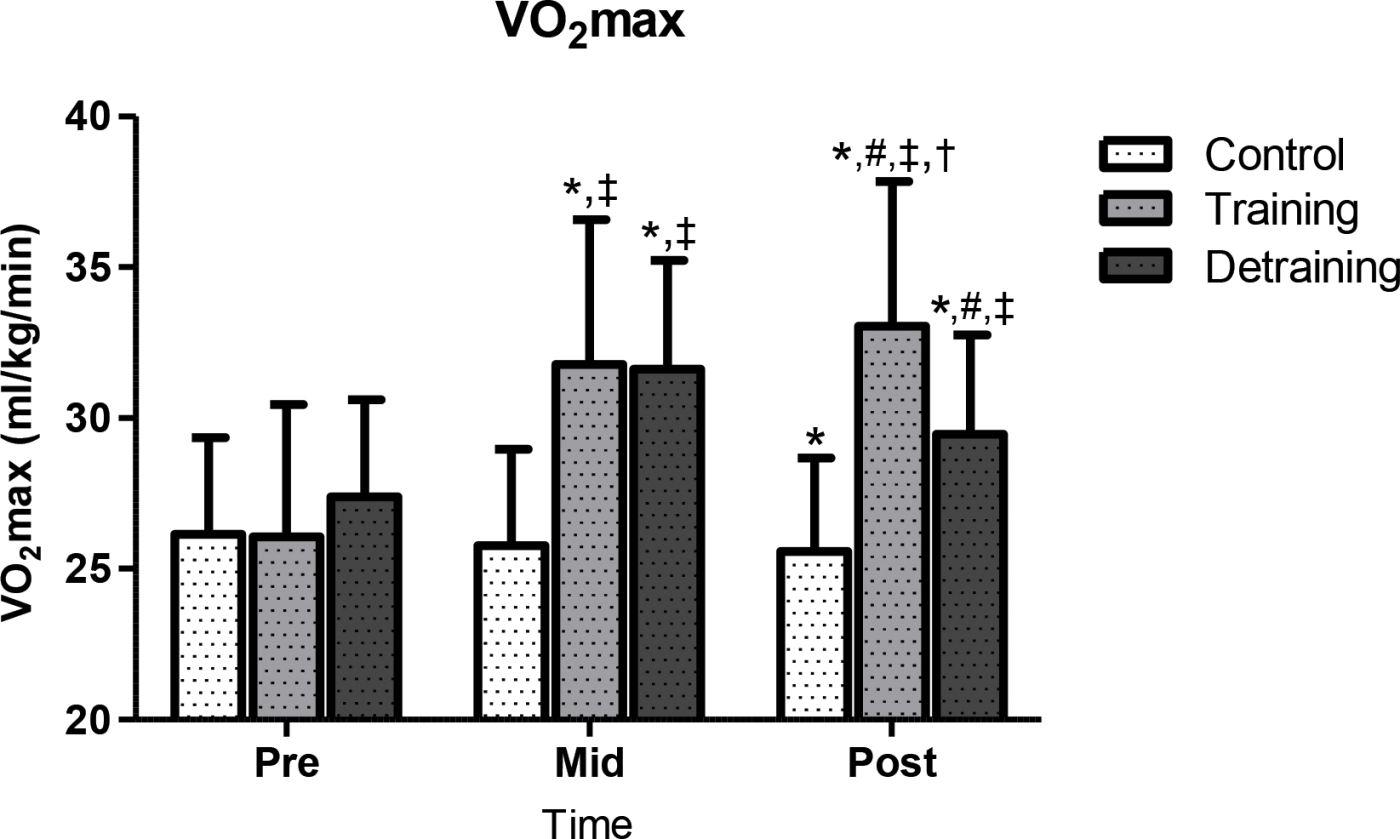
VO_2max_ changes during the experimental period. VO_2max_, maximal oxygen intake; *significant difference with baseline (p<0.05); ‡significant difference with the control group (p<0.05); #significant difference with previous time point (p<0.05); †significant difference between training and training-detraining groups (p<0.05).

**Fig 4:**
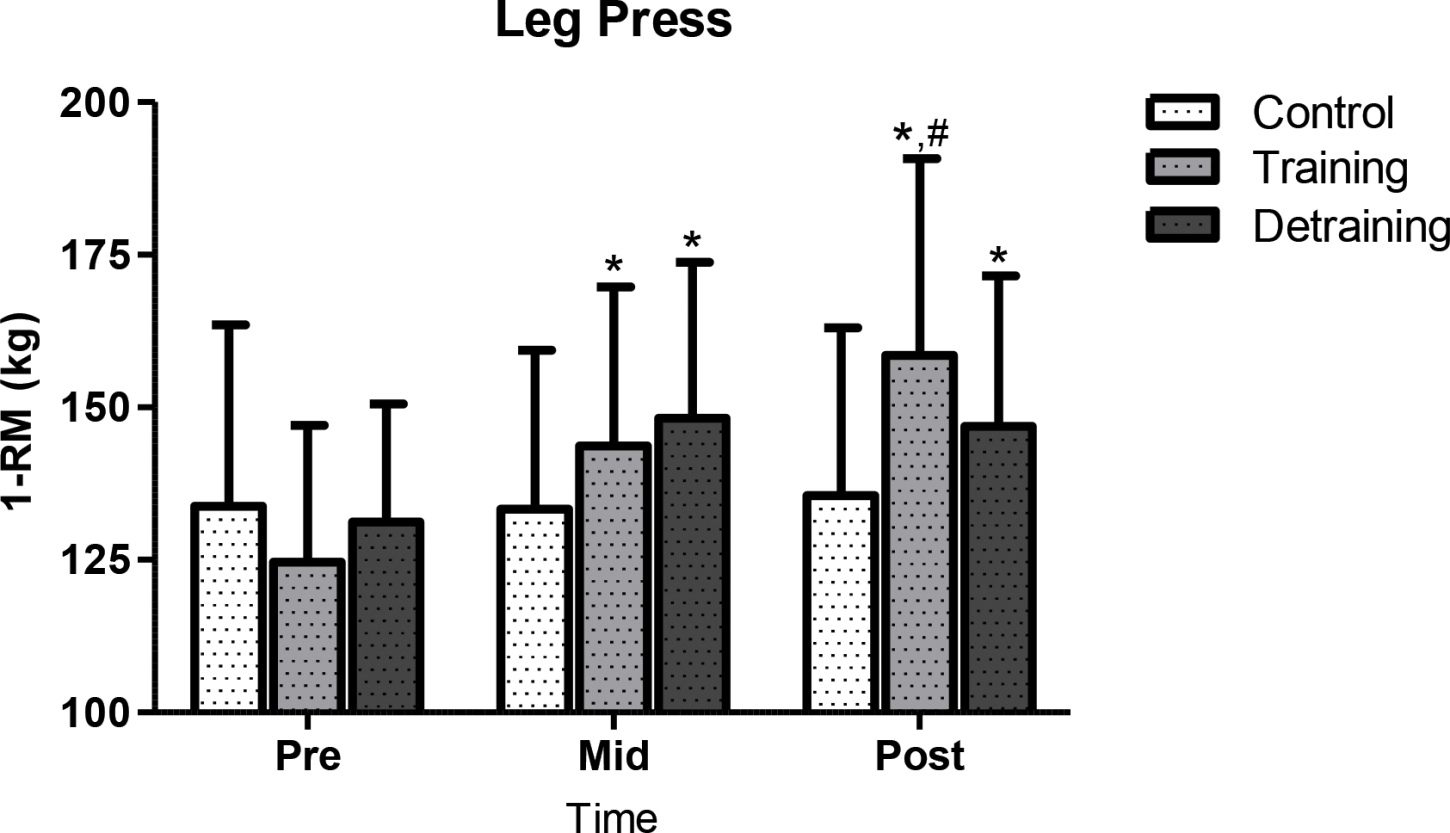
1RM changes during the experimental period. 1RM, one repetition maximum; *significant difference with baseline (p<0.05); #significant difference with previous time point (p<0.05).

Performance results are shown in Figs 3 and 4. In C, VO_max_ declined (p<0.013; CI = -0.43, 0.79; ES = 0.18) at 40 weeks (25.6 ± 3.1 ml·kg-1·min-1, -2%) compared to baseline (26.1 ± 3.2 ml·kg-1·min-1). In TR, VO_2max_ increased progressively from baseline (26.1 ± 4.4 ml·kg-1·min-1) to mid-training (31.8 ± 4.8 ml·kg-1·min-1; p<0.001; CI = -2.01, -0.40; ES = -1.20; +21.8%) and post-training (33.1 ± 4.8 ml·kg-1·min-1; p<0.001; CI = -1.00, -0.49; ES = -0.26; +26.8%). In TRD, VO_2max_ increased from baseline (27.4 ± 3.2 ml·kg-1·min-1) to mid-training (31.6 ± 3.6 ml·kg-1·min-1; p<0.001; CI = -2.01, -0.40; ES = -1.20; +15.3%) and decreased thereafter (29.5 ± 3.3 ml·kg-1·min-1; p<0.001; CI = -0.15, -1.37; ES = 0.61; -6.6%) but remained above pre-training levels (p<0.001; CI = -1.37, - 0.15; ES = -0.61; +7.7%). At mid-training, VO_2max_ demonstrated similar values in TR and TRD and they were both higher than C (C vs. TR: +23.3%, p<0.001; CI = -2.26, -0.74; ES = -1.50; C vs. TRD: +22.5%, p<0.001; CI = -2.50, -0.93; ES = -1.71). At 40 weeks, TR demonstrated higher VO_2max_ values than C and TRD (TR vs. C: +29.3%, p<0.001; CI = 1.08, 2.70; ES = 1.89; TR vs. TRD: +12.2%, p<0.05; CI = 0.08, 1.62; ES = 0.85) and TRD than C (p<0.012; CI = 0.46, 1.92; ES = 1.19; +15.2%). These VO_2max_ results were not affected by training-induced weight loss since similar differences were detected when VO_2max_ was expressed in both relative (mL/kg/min) and absolute (L/min) terms. Strength (1RM) in C remained unchanged throughout the study. In TR, 1RM increased progressively from baseline (124.6 ± 22.4 kg) to mid-training (143.7 ± 26.0 kg; p<0.001; CI = 1.53, 0.00; ES = -0.77; +15.3%) and post-training (158.5 ± 32.3 kg; p<0.001; CI = -1.24, 0.26; ES = -0.49; +27.2%) and from mid-training to post-training (p<0.05, +10.3%). In TRD, 1RM increased from baseline (131.4 ± 18.6 kg) to mid-training (148.2 ± 25.6 kg; p<0.001; CI = -1.52, 0.07; ES = -0.73; +12.8%) and decreased thereafter but remained above pre-training levels (146.9 ± 24.6 kg; p<0.001; CI = -0.10, 1.48; ES = 0.69; +11.8%).

Table 5 demonstrates changes in anthropometric variables, resting metabolic rate, physical activity, and energy intake. Body mass and BMI remained constant in C, decreased in TR at mid-training (body mass: p<0.001; CI: -0.38, 1.12, ES = 0.37; BMI: p<0.001; CI: -0.28, 1.23, ES = 0.48) and demonstrated a trend for further reduction at post-training (body mass: p = 0.092; CI: -0.67, 0.82; ES = 0.08; BMI: p<0.086; CI: -0.64, 0.84; ES = 0.10). In TRD, body mass and BMI decreased at mid-training (body mass: p<0.001; CI: -0.42, 1.08; ES = 0.33; BMI: p<0.001; CI: -0.44, 1.05; ES = 0.30) but increased following detraining (body mass: p<0.003; CI: -0.88, 0.60, ES = -0.14; BMI: p<0.004; CI: -0.87, 0.62; ES = -0.12) without reaching pre-training levels. When body mass changes were adjusted across intervention by daily caloric consumption and daily physical activity using an ANCOVA, it was revealed that body mass was reduced at mid-training (p<0.001; CI = -0.38, -1.12; ES = 0.37) and further decreased at post-training (p<0.04; CI = -0.67, -0.82; ES = 0.08) whereas in C and TRD results remained similar to those produced by the ANOVA. Body fat increased in C at 40 weeks (post- vs. pre-training: p<0.008; CI: -0.76, 0.45, ES = -0.15) but in TR and TRD it declined both at mid- (TR: p<0.001; CI: 0.28, 1.87, ES = 1.08; TRD: p<0.001; CI: -0.27, 1.24, ES = 0.49) and post-training (TR: p<0.001; CI: -0.44, 1.05, ES = 0.30; TRD: p<0.001; CI: -0.92, 0.57, ES = -0.17). Fat-free mass remained unchanged in C but increased at mid- (TR: p<0.001; CI: -1.07, 0.42, ES = -0.32; TRD: p<0.015; CI: -1.06, 0.43, ES = -0.32) and post-intervention (TR: p<0.001; CI: -1.20, 0.30, ES = -0.45; TRD: p<0.001; CI: -1.04, 0.45, ES = -0.29) in the other two groups. In C, waist circumference (p<0.059; CI: -0.93, 0.29; ES = -0.32) and hip circumference (p<0.012; CI: -0.92, 0.30; ES = -0.31), but not WHR, tended to increase at 40 weeks. Waist circumference, hip circumference and WHR decreased in TR at mid-training (waist circumference: p<0.001; CI: -0.09, 1.44; ES = 0.68; hip circumference: p<0.002; CI: -0.35, 1.15; ES = 0.40; WHR: p<0.059; CI: -0.93, 0.29; ES = -0.32) and remained above baseline at post-training (waist circumference: p<0.001; CI: -0.03, 1.50; ES = 0.74; hip circumference: p<0.001; CI: -0.31, 1.19; ES = 0.44; WHR: p<0.001; CI: 0.08, 1.63; ES = 0.86). In TRD, waist circumference, hip circumference and WHR decreased at mid-training (waist circumference: p<0.001; CI: 0.000, 1.53; ES = 0.77; hip circumference: p<0.031; CI: -0.47, 1.01; ES = 0.27; WHR: p<0.001; CI: -0.02, 1.51; ES = 0.74) and increased (waist circumference: p<0.001; CI: -1.23, 0.27; ES = -0.48; hip circumference: p<0.015; CI: -0.90, 0.59; ES = -0.16; WHR: p<0.001; CI: -1.16, 0.33; ES = -0.42) at 40 weeks without reaching pre-training values. Resting metabolic rate remained unaffected in C, increased progressively throughout the study in TR (mid training: p<0.05; CI: -1.30, 0.21; ES = -0.54; post-training: p<0.012; CI: -1.12, 0.37; ES = -0.37) whereas in TRD it increased at mid-training (p<0.001; CI: -1.43, 0,09; ES = -0.67) and decreased at post-training without reaching baseline values (p<0.001; CI: -0.10, 1.42; ES = 0.66). Daily physical activity and energy intake were similar in all groups and no changes were observed throughout the study.

**Table 5:** Changes of anthropometric and performance variables, resting metabolic rate, habitual physical activity, and energy intake during the experimental period.

	Baseline			20 weeks			40 weeks		
Variables	C	TR	TRD	C	TR	TRD	C	TR	TRD
Sedentary PA (min)	1205.5±88.4	1230.3±75.2	1203.8±80.1	1206.3±108.8	1230.7±70.9	1190.5±80.1	1200.8±232.1	1216.6±74.5	1202.4±83.1
Light PA (min)	198.5±78.1	179.1±74.9	191.8±66.5	196.9±102.0	175.9±71.3	204.6±69.4	156.0±101.6	186.0±72.2	193.6±63.5
Moderate PA (min)	34.9±13.1	29.6±8.8	42.4±20.7	35.8±12.1	31.4±9.1	42.7±18.7	34.9±12.5	35.1±7.8	38.5±19.5
Vigorous PA (min)	1.05±0.8	0.98±1.8	0.91±1.5	0.94±0.7	1.29±1.9	1.21±1.5	1.02±0.8	1.51±1.7	1.19±1.3
MVPA (min)	36.0±13.6	30.5±9.6	43.3±21.2	36.8±12.6	32.7±9.9	43.9±19.2	35.9±13.2	36.6±8.5	39.7±19.8
Steps·day^-1^	6399.7±1851.3	6330.7±1279.0	6870.0±2030.6	6370.4±1827.0	6363.9±1384.0	6933.7±1998.7	6351.0±1900.1	6536.8±1319.3	6748.5±1939.6
Kcal·day^-1^	182.2±73.6	158.9±60.0	214.1±88.6	182.3±70.6	164.9±58.3	215.4±84.8	183.5±71.3	181.4±60.0	183.1±67.5
METs·day^-1^	1.40±0.16	1.09±0.03	1.12±0.05	1.41±0.13	1.30±0.18	1.32±0.14	1.42±0.14	1.28±0.18	1.34±0.16
EI (kcal·day^-1^)	1829.9±193.9	1840.5±151.8	1839.6±230.8	1835.4±178.0	1807.4±163.3	1812.4±231.5	1834.1±158.0	1782.9±150.2	1807.2±201.3
Protein (%)	16.5±0.05	17.4±0.04	16.8±0.03	17.1±0.03	16.6±0.02	17.3±0.03	17.7±0.03	18.3±0.03	17.5±0.04
Carbohydrate (%)	58.8±0.05	57.5±0.04	57.7±0.03	56.8±0.03	58.7±0.04	58.1±0.03	57.9±0.03	56.0±0.03	58.8±0.03
Fat (%)	24.7±0.04	25.0±0.03	25.5±0.04	26.1±0.03	24.7±0.04	24.6±0.03	24.4±0.03	25.7±0.03	23.7±0.04
RMR (kcal·day^-1^)	1501.1±162.8	1451.6±145.4	1504.1±220.3	1507.6±150.9	1536.4±158.1^a^	1637.9±163.6^a^	1523.7±141.8	1597.9±160.9^b^^,^^c^	1524.9±170.7^b^
Body mass (kg)	80.2±8.9	78.0±9.9	78.2±7.8	80.4±7.7	74.2±10.3^a^	75.5±8.2^a^	80.9±7.7	73.4±10.0^c^	76.7±9.0^b^
Body height (m)	1.65±0.05	1.66±0.05	1.64±0.06	1.65±0.05	1.66±0.05	1.64±0.06	1.65±0.05	1.66±0.05	1.64±0.06
BMI (kg·m^-2^)	29.6±3.0	28.4±2.8	29.1±3.0	29.5±2.7	26.8±2.9^a^	28.1±3.4^a^	29.9±2.7	26.5±2.7^c^	28.6±3.5^b^
Body fat (%)	46.7±6.5	47.5±3.2	46.2±3.9	47.1±6.5	43.4±4.2^a^	43.8±5.5^a^	47.7±6.5^b^^,^^c^	42.0±4.5^b^^,^^c^	44.8±5.1^b^^,^^c^
Fat mass (kg)	37.4±6.5	37.3±6.7	36.3±6.0	37.8±6.2	32.5±7.1^a^	33.4±7.1^a^	38.6±6.5^b^^,^^c^	31.1±6.9^b^^,^^c^	34.6±7.3^b^^,^^c^
Fat-free mass (kg)	42.8±7.2	40.8±4.1	41.9±3.2	42.6±6.7	41.7±4.1^a^	42.1±3.3^a^	42.3±6.6	42.3±4.5^b^^,^^c^	42.1±3.6^b^^,^^c^
WCR (cm)	95.9±5.3	96.7±8.8	96.4±8.9	96.1±4.8	90.8±8.1^a^	89.3±9.0^a^	97.6±5.1^b^	90.1±8.7^c^	94.0±9.9^b^^,^^c^
HCR (cm)	110.3±6.5	110.9±6.5	110.9±7.2	111.0±6.1	108.0±7.6^a^	108.8±7.9^a^	112.2±5.6^b^^,^^c^	107.9±7.1^c^	110.0±7.1^b^
WHR	0.87±0.04	0.87±0.04	0.87±0.06	0.87±0.04	0.84±0.04^a^	0.82±0.07^a^	0.87±0.04	0.83±0.05^c^	0.85±0.07^b^
VO_2max_ (ml·kg-1·min-1)	26.1±3.2	26.1±4.4	27.4±3.2	25.8±3.2	31.8±4.8^a^	31.6±3.6^a^	25.6±3.1^a^	33.1±4.8^a^^,^^b^^,^^c^	29.5±3.3^a^^,^^b^^,^^c^
1RM Leg press (kg)	133.8±29.7	124.6±22.4	131.4±18.6	133.3±26.1	143.7±26.0^a^	148.2±25.6^a^	135.5±27.5	158.5±32.3^a^^,^^b^	146.9±24.6^a^

C, control group; TR, trained group; TRD, trained-detrained group; PA, physical activity; MVPA, moderate-to-vigorous physical activity; METs, metabolic equivalent of task; EI, energy intake; RMR, resting metabolic rate; BMI, body mass index; WCR, waist circumference; HCR, hip circumference; WHR, waist-to-hip ratio; VO_2_max, maximal oxygen intake; 1RM, one repetition maximum;

^a^denotes a difference between baseline and mid-training at p<0.05 or at p<0.01;

^b^denotes a difference between mid-training and post-training or detraining at p<0.05 or at p<0.01;

^c^denotes a difference between baseline and post-training or detraining at p<0.05 or at p<0.01.

**Fig 5:**
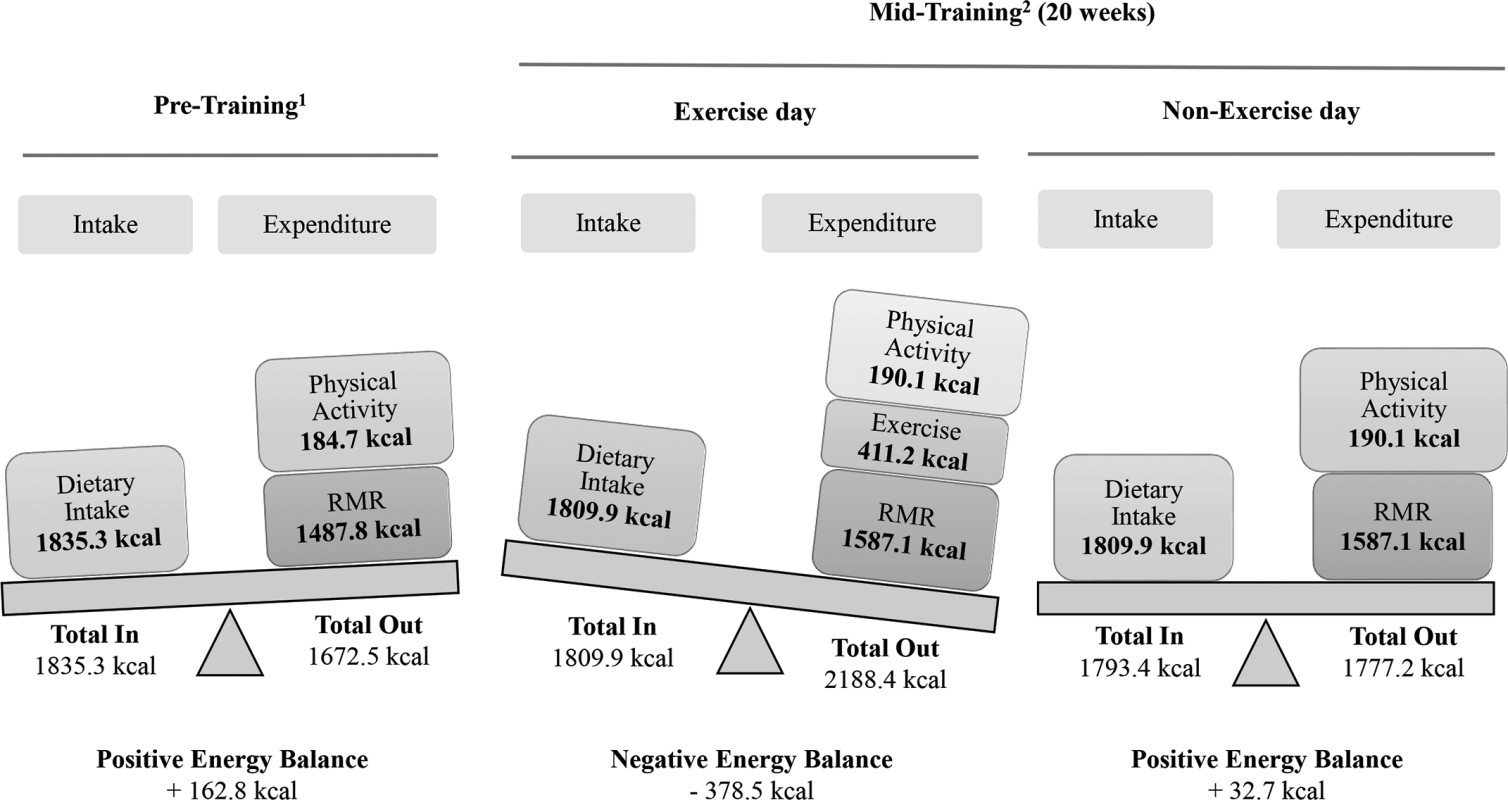
Changes in daily energy balance in the control and experimental groups at pre- and mid-training. RMR, resting metabolic rate; ^1^mean values for all groups; ^2^mean values for training and training-detraining groups.

**Fig 6:**
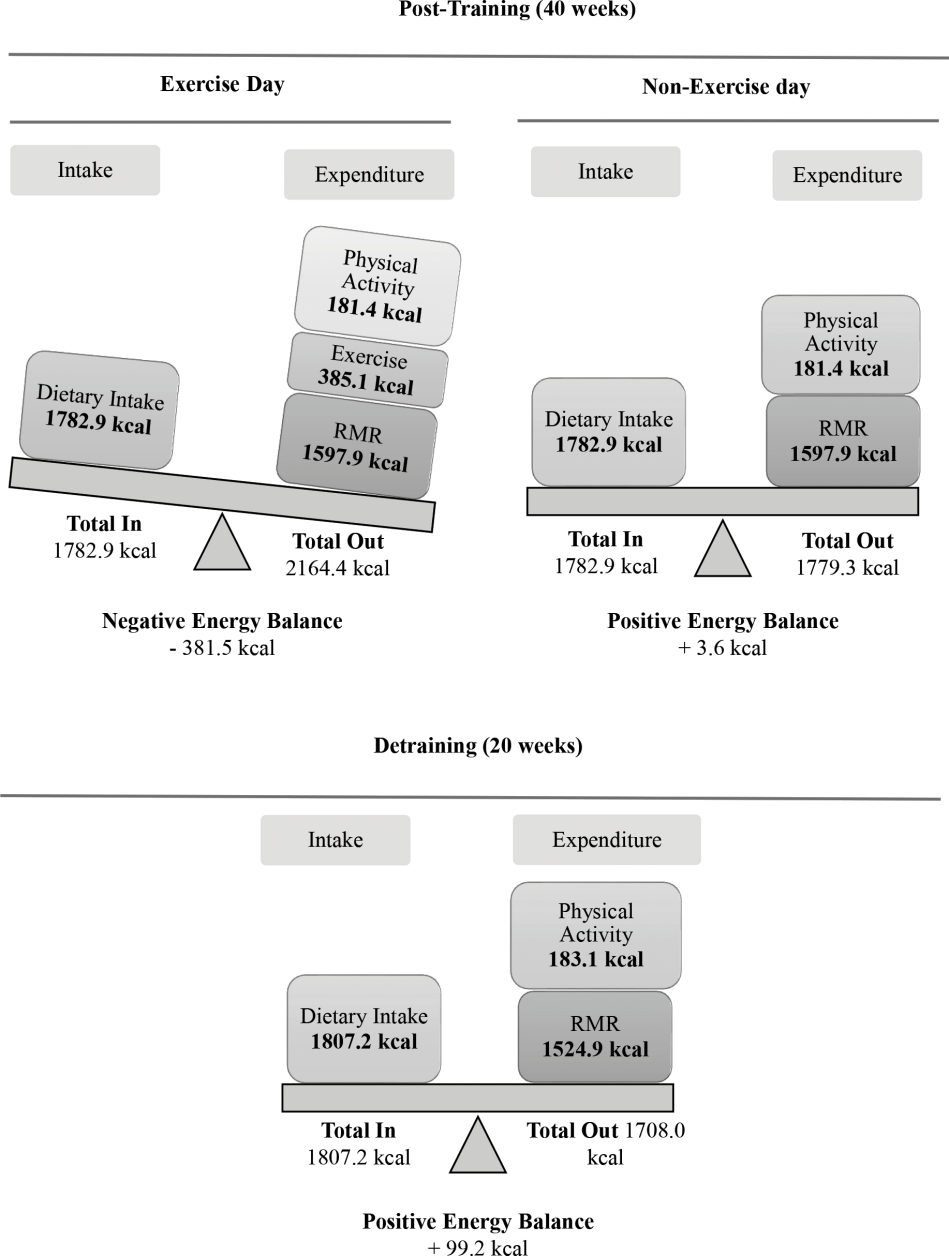
Changes in daily energy balance in the experimental groups at post-training and detraining. RMR, resting metabolic rate.

Figs 5 and 6 provide a schematic representation of energy balance at baseline, at mid-training and at post-training (or detraining) for an exercise and a non-exercise day (values are based on food kcal for energy intake and on the sum of kcal for resting metabolic rate, habitual physical activity and exercise for energy expenditure). Training augmented energy expenditure thus contributing to an increased overall energy deficit on a weekly basis.

## Discussion

A 10-month implementation of a circuit-type integrated neuromuscular small-group training program resulted in i) enhanced daily energy expenditure over energy intake thereby reducing body and fat mass; ii) increased strength and cardiovascular performance; and iii) a high adherence rate. Training-induced gains were attenuated but not lost following a 5-month detraining period.

In developed countries, 42–51% of Caucasian women aged 20–40 years are classified as overweight or obese [7], may gain 6–12 kg more than any other population group [8], and exhibit increased inactivity levels [9]. CINT alone reduced women’s body mass by ~6% after 10 months. This magnitude of weight loss is in line with AHA guidelines and coincides with reports of studies that utilized high-intensity, interval-type exercise protocols in overweight/obese adults [6,18]. These findings confirm that weight is only modestly reduced when exercise is the sole weight management intervention [40]. Continuous endurance training induces a weight loss of smaller magnitude [11] and only when applied at higher dosages (13–26 MET-hours/week) it elicits a more pronounced loss [12,13]. Losses of >5% are seen in response to protocols of combined diet and exercise or to continuous endurance training of very high volume (≥26 MET-hours/week) [6,12]. CINT induced a ~6% weight loss with a metabolic overload of only 5–12 MET-hours/week. A wright loss of similar magnitude is associated with clinically meaningful health benefits [6]. Although overweight adults usually equilibrate after 6 months of weight loss intervention demonstrating a plateau and a gradual weight regain over time [6], CINT maintained weight loss over a 10-month period.

CINT decreased body fat by ~5.5%, a reduction usually seen with tri-weekly continuous endurance training protocols of similar duration [11]. HIIT-type protocols are effective in reducing visceral and subcutaneous fat in overweight women [13,41,42,43]. Similar rates of fat loss have been seen in response to exercise dosages of ~10 MET-hours/week [42,44], as in CINT. The increase in fat-free mass (1.2–3.4%) is attributed to the protocol’s resistance exercise component [11]. The decline in waist-to-hip ratio indicates body fat redistribution which is usually observed following a 5-month high-volume endurance training [45]. CINT prevented inactivity-induced weight (0.7 kg) and fat (1%) gain seen in controls over a 10-month period.

Weight loss represents a deficit between total daily energy intake and expenditure [46]. Energy expenditure is related to energy cost for resting metabolic rate, movement, digestion/metabolism and thermogenesis [47]. Exercise training increases energy expenditure and reduces body fat by upregulating intracellular signaling cascades in adipose tissue that promote thermogenesis and lipolysis [3,48,49,50]. Although energy expenditure associated with thermogenesis/digestion/metabolism (≤10%) was not measured, participant’s daily energy balance was estimated by subtracting the energy expenditure associated with resting metabolic rate and movement from the energy intake through diet (Figs 5 and 6).

Daily energy intake remained unaltered during the study since participants were asked to follow an isocaloric diet. On a non-exercise day, energy expenditure is the energy cost of habitual physical activity and resting metabolic rate. The former remained unchanged throughout the study while the latter increased by 6%-10% (Figs 5 and 6). Usually, weight loss following a period of endurance exercise and isocaloric feeding results in reduced resting metabolic rate by ~7% which may compromise exercise-induced energy deficit [46]. However, CINT-induced elevation of fat-free mass probably prevented such a response and contributed to the elevation of resting metabolic rate [32,48], a response also seen with resistance training [42]. The rise in fat-free mass is also evidenced by the marked strength increase (~27%), a response typically seen with HIIT-type programs due to increased activation of muscle fibers, mitochondrial biogenesis and glucose transport [25]. The substantial increase (~25%) of VO_2max_ provides further supports that CINT induced favorable mitochondrial adaptations similarly to other HIIT protocols [18]. The rise in excess post-exercise oxygen consumption seen here and in previous studies may further explain the augmented resting metabolic rate during the post-exercise period [18,48,51].

On an exercise day, energy expenditure is primarily associated with the intensity and/or volume of the exercise session [32,52]. The exercise energy cost/session ranged from ~165 kcal (phase 1) to >400 kcal (phases 3 and 4), which is well over that reported for HIIT-type programs utilizing 30-s sprints (175 kcal/session) or circuit resistance training (≤250 kcal/session) and almost equal with that induced by traditional moderate, 30-min continuous endurance training in obese adults [32,48,53]. Interval-type protocols upregulate adipose tissue lipolysis and elevate post-exercise oxygen consumption that may further increase daily energy expenditure [18,48,51].

After 5 months of training (Fig 5), CINT induced a deficit of ~380 kcal/day on an exercise day (three days/week) and a small caloric deficit of ~30 kcal/day on a non-exercise day (four days/week). Daily this is translated to a deficit of ~140 kcal which by far exceeds the energy surplus estimated at baseline (+160 kcal/day). This is further translated to a deficit of ~1,000 kcal/week for the training groups at mid-training whereas the controls had a surplus of ~1,150 kcal/week. Over a 40-week training period (Fig 6), the overall energy deficit is estimated at ~42,700 or roughly a loss of ~5.7 kg of fat (i.e. ~0.14 kg/week) which is close to the measured loss of fat mass (~6 kg). Similar rates of fat loss have been reported in response to long-term implementation of HIIT-type programs in obese women [54] probably due to enhanced catecholamine-mediated lipolysis and fat oxidation, especially in visceral fat [18,48].

Cessation or frequent interruptions of training are common in anti-obesity exercise interventions causing metabolic decompensation that leads to weight regain [55]. Detraining resulted in a regain of body and fat mass (1.7% and 3.7%, respectively), a decline of fat-free mass (2.4%) and consequently in reduced energy deficit without reaching pre-training values. During the same period, VO_2max_ decreased by ~7% indicating a metabolic decompensation. Similar results were seen with older adults who were subjected to a 6-month detraining following training with high-intensity resistance exercise [32]. High-intensity exercise may prevent a significant drop of resting metabolic rate during detraining resulting in only a modest regain of body and fat mass [32]. Fat regain during detraining is associated with decreased lipolysis and increased triacylglycerol synthesis due to elevated glucose uptake by adipose tissue [56].

CINT maintained a relatively high intensity as evidenced by the gradual rise of mean heart rate (72–87%), blood lactate (8–12 mM), RPE (13–16), METs (5–7) and VO_2_ (18–24 mL/kg/min) over a 10-month implementation. The intensity reached levels relatively higher than those usually seen during intense interval running (79%) that resulted in reduced body fat [18]. CINT’s total duration/session was 18–36 min (net exercise time of 6–24 min) and work-to-rest ratio ranging from 1:2 to 2:1 as most HIIT protocols that effectively reduced body mass [41,43]. Continuous endurance training elicit modest (~2–3 kg), large (~5–7.5 kg) or even larger (> 8 kg) weight losses when they exceed 150, 250 and 400 min/week, respectively [11,13]. In contrast, CINT and other HIIT-type protocols elicit similar or even larger weight loss spending ≤100 min/week [41,43]. This is important because high weekly exercise volumes are associated with greater attrition and lower compliance rates [12]. Results corroborate previous reports suggesting that prolonged interventions induce a greater fat mass reduction in obese adults when compared to short-lived protocols [6,11]. Although a higher frequency (5 vs. 3 days/week) may be more effective in reducing fat mass [57], CINT and other HIIT protocols produce are effective using a lower frequency [41,43]. The small-group training model using bodyweight exercises represents a promising trend in the fitness industry worldwide [58] because it is appealing to clients and time- and cost-effective, although not evidence-based yet.

Anti-obesity programs employing lifestyle interventions (i.e., exercise and/or nutrition) in overweight/obese adults usually exhibit limited success rates mainly due to low adherence/attendance (20–80%), especially in women [17,59]. For reasons not currently known, CINT had a 6% attrition, as defined by Miller et al. [55], and 94% attendance.

This study included only previously inactive overweight/obese premenopausal Caucasian females which limits the ability to extrapolate the findings to males, other age groups or diverse types of populations. Males, compared to females, tend to lose more body fat and increase at a greater extent their maximal oxygen consumption in response to exercise interventions whereas in females the rate of fat loss seem to depend on weekly duration of exercise protocols [59]. Although the duration of CINT was 10 months, participants in lifestyle-based anti-obesity interventions reach a peak weight loss within 6 months of treatment and tend to regain weight thereafter with 50% of them returning to their pre-intervention body mass after ~5 years [60]. According to Wing and Hill, intentional weight loss should be maintained for at least one year [61]. As such, future studies should investigate the effectiveness of CINT-type protocols on weight loss for more prolonged periods. Cessation or interruption of training is a widespread problem in weight loss programs [55]. Most studies that examined the effects of training cessation on anthropometric, physiological and biochemical markers in overweight/obese adults applied a 4-week detraining period producing different results [62–64]. In this study, a 5-month detraining period was applied to investigate the maintenance rates of training gains. It is possible that a more prolonged detraining period would have caused a greater rate of deterioration of training-induced adaptations.

### Conclusions

An injury-free, high-intensity, interval exercise training program using whole-body resistance exercises and cardiovascular-type activity, of limited time commitment, organized in a small-group setting, induced favorable adaptations in body and fat mass of overweight/obese women. CINT-type protocols utilize only bodyweight exercises that promote body mass and fat loss and improve both strength and endurance performance. These changes are attributed to an increase in resting metabolic rate and fat-free mass that resulted in increased energy expenditure. Detraining-induced weight/fat regain was limited suggesting that this type of exercise training may promote long-term maintenance of weight loss in overweight adults.

## Supporting information

S1 Protocol Study protocol. (PDF)

S2 Protocol Study protocol in Greek. (PDF)

S1 CONSORT Checklist CONSORT 2010 checklist. (PDF)

S1 Data Study data. (XLSX)
