# Correction: High intensity, circuit-type integrated neuromuscular training alters energy balance and reduces body mass and fat in obese women: A 10-month training-detraining randomized controlled trial

**DOI:** 10.1371/journal.pone.0240945

**Published:** 2020-10-13

**Authors:** Alexios Batrakoulis, Athanasios Z. Jamurtas, Kalliopi Georgakouli, Dimitrios Draganidis, Chariklia K. Deli, Konstantinos Papanikolaou, Alexandra Avloniti, Athanasios Chatzinikolaou, Diamanda Leontsini, Panagiotis Tsimeas, Nikolaos Comoutos, Vassilios Bouglas, Maria Michalopoulou, Ioannis G. Fatouros

There are a number of errors in Fig 1, “CONSORT diagram of the study.” Please see the complete, correct Fig 1 here.

**Fig 1:**
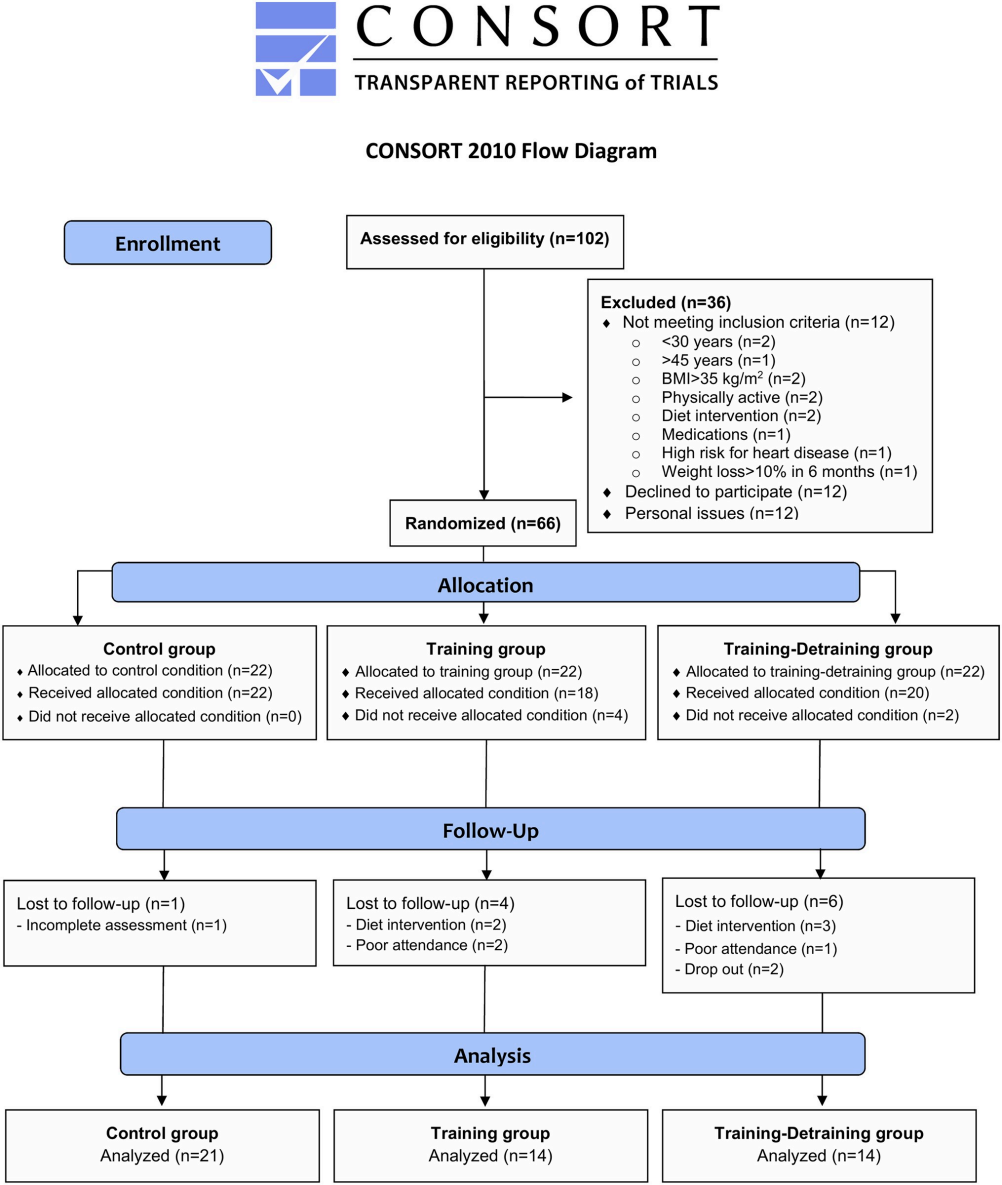
CONSORT diagram of the study.

In the Participants and research design subsection of the Materials and methods, there are errors in the fifth sentence of the second paragraph. The correct sentence is: One hundred and two females were interviewed, 66 were recruited (12 were not interested in participating, 12 did not meet the inclusion/exclusion criteria and 12 were excluded due to personal issues) and 49 completed it [data from 11 women were not used because of altered energy intake during the study (5 women), poor attendance (3 women), drop out (2 women), and failure to participate in all measurements (1 woman)] (Fig 2).
