# Supplementary material for: High intensity, circuit-type integrated neuromuscular training alters energy balance and reduces body mass and fat in obese women: A 10-month training-detraining randomized controlled trial
Source: PLoS One. 2018 Aug 23;13(8):e0202390. doi: 10.1371/journal.pone.0202390 (PMC6107179; doi:10.1371/journal.pone.0202390)
Supplement: S1 Protocol — (PDF) [file pone.0202390.s001.pdf]

STUDY PROTOCOL

To: Ethics Committee of the Department of Physical Education and Sports Science (DPESS) of the University of Thessaly (UTh).

Title: The effects of a circuit-type integrated neuromuscular training on body composition, body weight and energy balance in sedentary overweight/obese women.

Advisory Committee:
Athanasios Jamurtas, Associate Professor, University of Thessaly
Ioannis Fatouros, Associate Professor, University of Thessaly
Nikolaos Zourbanos, Assistant Professor, University of Thessaly

Relationship with the study programs of the DPESS at UTh:
Master's Thesis of Alexios Batrakoulis as a part of the requirements for the degree of Master Science (MSc) in Exercise and Health Graduate Program of DPESS at UTh.

Submitted on May 20, 2015

Purpose of the study

The purpose of this investigation will be the determination of the effects of a functional circuit training protocol with whole-body exercises using alternative modalities on body mass, body composition and energy balance of previously inactive, overweight/obese women. The initial hypothesis will be that this type of training and its subsequent detraining would not alter body mass, body fat and energy balance of overweight women.

Importance of the study

Body weight training, high-intensity interval training (HIIT), exercise for weight loss, personal and group personal raining, functional fitness, and circuit training are currently considered some of the top worldwide trends in the commercial, corporate, clinical, and community landscapes within the health and fitness industry (Thompson, 2016). In addition, the most recent epidemiology findings and future predictions by the World Health Organization (WHO) regarding the prevalence of overweight and obesity adult population indicate that more than one out of two in Europe and two out of three worldwide are and will be maintaining overweight (WHO, 2014). On the other side, physical inactivity rates have grown immensely worldwide (Hallal et al., 2012) although regular exercise induces many positive changes in health by lowering the risk factors for several chronic diseases. (Blair et al., 1996).

Introduction and literature review

High-intensity interval training (HIIT), circuit weight training (CWT) and functional fitness training (FFT) are considered some of the top fitness trends worldwide, time-effective, safe and efficient exercise modalities for general population (Thompson, 2014). According to the ACSM's position statement, HIIT, CWT, and FFT induce the improvement of cardiovascular endurance, musculoskeletal health, and functional capacity in healthy adults (Garber et al., 2011). Additionally, fitness professionals who are interested in providing clients with the optimal results in less possible time, they always seek some innovative training methods and protocols

(Paton & Hopkins, 2005) that are able to be more efficient and time-effective than some similar traditional options (Jones, Parker, & Cortes, 2011).

HIIT consists of alternatively repeated work intervals of high-intensity exercise with rest intervals of low-intensity exercise or complete rest and has become an increasingly popular exercise modality due to its potentially large effects on aerobic and anaerobic capacity, and short time requirement (Cress, Porcari, & Foster, 2015; Foster et al., 2015; Kilpatrick, Jung, & Little, 2014). HIIT is not only considered a time-efficient approach compared to traditional CET (Foster et al., 2015) but it has also been shown to improve physical fitness components within a short time period compared to the traditional CET (Hazell, MacPherson, Gravelle, & Lemon, 2010; Ziemann et al., 2011) and induces physiological adaptations that improve both health (Gibala, Little, Macdonald, & Hawley, 2012; Gremeaux et al., 2012) and performance (Gillen & Gibala, 2014) of healthy adults. Many researchers have already studied the effects of HIIT in healthy, sedentary overweight or obese adults finding improvements in aerobic capacity (Lunt et al., 2014; Trilk, Singhal, Bigelman, & Cureton, 2011), body composition (Gremeaux et al., 2012; Hazell, Hamilton, Olver, & Lemon, 2014; Trapp, Chisholm, Freund, & Boutcher, 2008), insulin resistance (Whyte, Gill, & Cathcart, 2010), RMR (Kelly, King, Goerlach, & Nimmo, 2013), hormones that increase metabolism (Racil et al., 2013), lipid profile (Miller et al., 2014), and skeletal muscle metabolism (Schjerve, 2008).

Recent evidence suggests that high-intensity intermittent circuit-style resistance training protocols may be considered more efficient than traditional steady state sustained-effort aerobic work or traditional resistance training programs (Klika & Jordan, 2013). Furthermore, this training method induces decrease of body fat in physically inactive middle-aged women with a normal BMI (Ferreira et al., 2010) and improves muscular strength, body composition, waist-to-hip ratio (WHR), waist circumference (WC), and blood lactate (BLA) production in overweight middle-aged untrained (Paoli et al., 2010). Applying the CWT in overweight middle-aged it has been observed a significant decrease on health markers, such as hypertension and blood lipids in comparison with the traditional endurance training and low-intensity circuit training (Paoli et al., 2013). Additionally, CWT may slightly increase resting energy expenditure (REE) and decrease respiratory rate (RR) 22 hours post-workout (Paoli et al., 2012).

The combination of cardiorespiratory training and resistance training is a basic methodology in exercise programming for physical performance and health of middle-aged and older adults with no risk factors for chronic diseases (Kraemer, Ratamess, & French, 2002; Takeshima et al., 2004; Wood et al., 2001). Moreover, CWT contributes to the decrease in risk factors for metabolic diseases and coronary artery disease (CAD) and improves significantly lipid profile (Fett, Fett, & Marchini, 2009). Furthermore, three different CWT protocols that included resistance training exercises and steady state aerobic or/and HIIT induce significant acute effects on BLA, heart rate (HR), and rating of perceived exertion (RPE) in recreationally active women (Skidmore, Jones, Blegen, & Matthews, 2012).

There is a research gap in the literature regarding the FT and its effects to physiological and metabolic adaptations in healthy adults compared to the traditional resistance training. There are only few studies that investigate chronic adaptations to body composition, physical performance, health, and wellness, and especially in sedentary overweight or obese individuals. More specifically, this type of training induces significant improvements in functional capacity (de Vreede, Samson, van Meeteren, Duursma, & Verhaar, 2005; Milton, Porcari, Foster, & Udermann, 2008;

Whitehurst, Johnson, Parker, Brown, & Ford, 2005) and body composition (Wiszomirska, Krynicki, Kaczmarczyk, & Gajewski, 2014) in elderly while improves musculoskeletal fitness health and flexibility in young adults (Kibele & Behm, 2009; Weiss et al., 2010). Moreover, it is suggested that FT increases adherence to regular exercise and engagement in structured training programs (Williams, Hendry, France, Lewis, & Wilkinson, 2007). In addition, high-intensity group fitness programs seem to be more attractive to the overweight and obese participants rather than traditional aerobics classes or traditional resistance training machine-based workouts. According to these research findings, participants who were engaged in this exercise modality improved significantly enjoyment, adherence, and intentions to systematic exercise experience. (Heinrich, Patel, O'Neal, & Heinrich, 2014).

Methods

Ethics statement

Participants signed a consent form after they were informed of all risks, discomforts and benefits involved in the study. Procedures were in agreement with the 1975 Declaration of Helsinki, as revised in 2000, and approval was granted by the institutional ethics committee of the Department of Physical Education and Sports Sciences of the University of Thessaly.

Participants and research design

The main goal of this investigation will be the evaluation of the efficacy of a circuit integrated neuromuscular training protocol with whole-body exercises using alternative modalities on energy balance of obese women and not to compare it with other exercise approaches used for weight management. A controlled, randomized, three-group, repeated-measures design will be employed. A preliminary power analysis will be completed in advance in order to be suggested the required sample size for this study. All participants will be asked to follow an isocaloric diet (based on RMR measurements and habitual physical activity). A Registered Dietitian will provide instructions on how to adapt to a weight maintenance diet (55-60% carbohydrate, 15-20% protein, 20%-25% fat), in the form of nutritional equivalents during an initial adaptation period when body mass will be monitored to verify the accuracy of the assigned energy approach (it will be re-adjusted at 20 weeks). To measure caloric intake, participants will submit 7-day diet recalls that will be analyzed by a Registered Dietitian using a nutritional analysis software. Participants will be instructed to maintain the same feeding pattern during the entire study.

Inclusion criteria

Participants will be recruited using fliers (posted in the local community), social media and by word of mouth. Women will be participating in the study should: (a) be inactive (<7,500 steps·day-1; VO2max <30 ml·kg-1·min-1; accelerometry-based moderate-to-vigorous physical activity <30 min·day-1), b) be healthy, premenopausal and aged 30-45 years, c) be overweight or obese class 1 [Body Mass Index (BMI) 25.1-34.9], d) have medical clearance for strenuous physical training, e) be non-smokers for ≥6 months before the study, f) not follow a diet intervention or use nutritional supplements/medications before (≥6 months) and during the study, g) have no weight loss greater >10% of body mass before (≤6 months) the study and h) have no symptoms of depression.

Exclusion criteria

Participants will be excluded from the study if: a) the adherence rate will be ≤80% of total exercise sessions, and i) they adhered to a nutritional intervention during the study.

Exercise intervention

A supervised small-group (5-10 women/session) circuit integrated neuromuscular training (CINT) protocol will be performed three times/week (with a 48-hour recovery between sessions), with the use of asynchronous music, for 40 weeks. During the first 20 weeks, training will be divided in three phases characterized by a progressive increase in exercise intensity and volume. During the second 20 weeks (phase 4), training will maintain the intensity and volume of phase 3 but the work-to-rest ratio will be varied bi-weekly. Exercises will incorporate fundamental movement patterns using bodyweight as resistance or adjunct portable modalities. Each session will include a 10-min warm-up (low-intensity endurance exercise, stretching exercises and mobility exercises) and a 5-min cool-down period (walking/stretching exercises). Exercises (~8-12/session) will be performed in timed circuit fashion using working or resting intervals of a prescribed time. Participants will perform as many repetitions as possible at each station with proper form at a controlled, moderate speed. Each session will be consisted of alternate stations emphasizing cardiovascular, resistance and neuromotor exercises. For resistance-type exercises, participants will be encouraged to use a comfortable resistance at the beginning of the study while progressing to heavier loads that will allow them to complete the desired exercise duration at each station. Heart rate will be monitored (Polar Team Solution) throughout each session and mean and maximal heart rate will be recorded. Participants will be guided to keep an intensity >75% of maximal heart rate. Rates of perceived exertion will be recorded for each round and mean exertion will be calculated. Exercise intensity will be calculated as mean heart rate (percentage of maximal heart rate obtained during VO_2max testing), percentage (%) of heart rate reserve, RPE, % of VO_2peak, metabolic equivalents of task (METs), and blood lactate accumulation for all participants.

Data collection

Anthropometric measurements will include height, body mass, Body Mass Index (BMI) and waist-to-hip ratio (WHR). Body composition will be measured by a whole-body dual-energy X-ray absorptiometry scanner (DXA). Strength (one repetition maximal, 1RM) will be measured bilaterally on a horizontal leg press machine using standard procedures while VO_2peak will be assessed during a graded maximal exercise testing (GXT) using the modified Balke protocol on a treadmill. Seven-day habitual physical activity level will be determined via accelerometry and the duration of inactivity and light, moderate, vigorous activity, steps/day and energy expenditure will be recorded. Participants will be encouraged to maintain their usual daily physical activity throughout the study. For the resting metabolic rate (RMR) assessment, resting VO_2/CO_2 will be measured in the morning (07.00-09.00) after an overnight fast using an open-circuit indirect calorimeter with a ventilated hood system while the 24-hour RMR will be calculated using the Weir equation.

References

de Vreede, P.L, Samson, M., van Meeteren, N.L.U., Duursma, S.A., & Verhaar, H.J.J. . (2005). Functional tasks exercise versus resistance strength exercise to improve daily function in older women: A randomized

controlled trial. *Journal of American Geriatric Society, 53*(1), 2-10.

Ferreira, F. C., de Medeiros, A. I., Nicioli, C., Nunes, J. E., Shiguemoto, G. E., Prestes, J., . . . Perez, S. E. (2010). Circuit resistance training in sedentary women: body composition and serum cytokine levels. *Appl Physiol Nutr Metab, 35*(2), 163-171. doi: 10.1139/H09-136

Fett, C. A., Fett, W. C., & Marchini, J. S. (2009). Circuit weight training vs jogging in metabolic risk factors of overweight/obese women. *Arq Bras Cardiol, 93*(5), 519-525.

Garber, C. E., Blissmer, B., Deschenes, M. R., Franklin, B. A., Lamonte, M. J., Lee, I. M., . . . American College of Sports, Medicine. (2011). American College of Sports Medicine position stand. Quantity and quality of exercise for developing and maintaining cardiorespiratory, musculoskeletal, and neuromotor fitness in apparently healthy adults: guidance for prescribing exercise. *Med Sci Sports Exerc, 43*(7), 1334-1359. doi: 10.1249/MSS.0b013e318213fefb

Gibala, M. J., Little, J. P., Macdonald, M. J., & Hawley, J. A. (2012). Physiological adaptations to low-volume, high-intensity interval training in health and disease. *J Physiol, 590*(Pt 5), 1077-1084. doi: 10.1113/jphysiol.2011.224725

Gillen, J. B., & Gibala, M. J. (2014). Is high-intensity interval training a time-efficient exercise strategy to improve health and fitness? *Appl Physiol Nutr Metab, 39*(3), 409-412. doi: 10.1139/apnm-2013-0187

Hazell, T. J., Hamilton, C. D., Olver, T. D., & Lemon, P. W. (2014). Running sprint interval training induces fat loss in women. *Appl Physiol Nutr Metab, 39*(8), 944-950. doi: 10.1139/apnm-2013-0503

Heinrich, K. M., Patel, P. M., O'Neal, J. L., & Heinrich, B. S. (2014). High-intensity compared to moderate-intensity training for exercise initiation, enjoyment, adherence, and intentions: an intervention study. *BMC Public Health, 14*, 789. doi: 10.1186/1471-2458-14-789

Jones, M. T., Parker, B. M., & Cortes, N. (2011). The effect of whole-body vibration training and conventional strength training on performance measures in female athletes. *J Strength Cond Res, 25*(9), 2434-2441. doi: 10.1519/JSC.0b013e31822817cf

Kelly, B., King, J. A., Goerlach, J., & Nimmo, M. A. (2013). The impact of high-intensity intermittent exercise on resting metabolic rate in healthy males. *Eur J Appl Physiol, 113*(12), 3039-3047. doi: 10.1007/s00421-013-2741-5

Kibele, A., & Behm, D. G. (2009). Seven weeks of instability and traditional resistance training effects on strength, balance and functional performance. *J Strength Cond Res, 23*(9), 2443-2450. doi: 10.1519/JSC.0b013e3181bf0489

Kraemer, W. J., Ratamess, N. A., & French, D. N. (2002). Resistance training for health and performance. *Curr Sports Med Rep, 1*(3), 165-171.

Lunt, H., Draper, N., Marshall, H. C., Logan, F. J., Hamlin, M. J., Shearman, J. P., . . . Frampton, C. M. (2014). High intensity interval training in a real world setting: a randomized controlled feasibility study in overweight inactive adults, measuring change in maximal oxygen uptake. *PLoS One, 9*(1), e83256. doi: 10.1371/journal.pone.0083256

Marx, J. O., Ratamess, N. A., Nindl, B. C., Gotshalk, L. A., Volek, J. S., Dohi, K., . . . Kraemer, W. J. (2001). Low-volume circuit versus high-volume periodized resistance training in women. *Med Sci Sports Exerc, 33*(4), 635-643.

Miller, M. B., Pearcey, G. E., Cahill, F., McCarthy, H., Stratton, S. B., Noftall, J. C., . . . Button, D. C. (2014). The effect of a short-term high-intensity circuit training program on work capacity, body composition, and blood profiles in sedentary obese men: a pilot study. *Biomed Res Int, 2014*, 191797. doi: 10.1155/2014/191797

Milton, D., Porcari, J. P., Foster, C., & Udermann, B. (2008). The effect of functional exercise training on functional frtness levels of older adults. *Gundersen Lutheran Medical Journal, 5*(1), 1-8.

Paoli, A., Moro, T., Marcolin, G., Neri, M., Bianco, A., Palma, A., & Grimaldi, K. (2012). High-Intensity Interval Resistance Training (HIRT) influences resting energy expenditure and respiratory ratio in non-dieting individuals. *J Transl Med, 10*, 237. doi: 10.1186/1479-5876-10-237

Paoli, A., Pacelli, F., Bargossi, A. M., Marcolin, G., Guzzinati, S., Neri, M., . . . Palma, A. (2010). Effects of three distinct protocols of fitness training on body composition, strength and blood lactate. *J Sports Med Phys Fitness, 50*(1), 43-51.

Paoli, A., Pacelli, Q. F., Moro, T., Marcolin, G., Neri, M., Battaglia, G., . . . Bianco, A. (2013). Effects of high-intensity circuit training, low-intensity circuit training and endurance training on blood pressure and lipoproteins in middle-aged overweight men. *Lipids Health Dis, 12*, 131. doi: 10.1186/1476-511X-12-131

Paton, C. D., & Hopkins, W. G. (2005). Combining explosive and high-resistance training improves performance in competitive cyclists. *J Strength Cond Res, 19*(4), 826-830. doi: 10.1519/R-16334.1

Racil, G., Ben Ounis, O., Hammouda, O., Kallel, A., Zouhal, H., Chamari, K., & Amri, M. (2013). Effects of high vs. moderate exercise intensity during interval training on lipids and adiponectin levels in obese young females. *Eur J Appl Physiol, 113*(10), 2531-2540. doi: 10.1007/s00421-013-2689-5

Skidmore, B. L., Jones, M. T., Blegen, M., & Matthews, T. D. (2012). Acute effects of three different circuit weight training protocols on blood lactate, heart rate, and rating of perceived exertion in recreationally active women. *J Sports Sci Med, 11*(4), 660-668.

Takeshima, N., Rogers, M. E., Islam, M. M., Yamauchi, T., Watanabe, E., & Okada, A. (2004). Effect of concurrent aerobic and resistance circuit exercise training on fitness in older adults. *Eur J Appl Physiol, 93*(1-2), 173-182. doi: 10.1007/s00421-004-1193-3

Trilk, J. L., Singhal, A., Bigelman, K. A., & Cureton, K. J. (2011). Effect of sprint interval training on circulatory function during exercise in sedentary, overweight/obese women. *Eur J Appl Physiol, 111*(8), 1591-1597. doi: 10.1007/s00421-010-1777-z

Waller, M., Miller, J., & Hannon, J. (2011). Resistance Circuit Training: Its Application for the Adult Population. *Strength Cond J, 33*(1), 16-22. doi: 10.1519/SSC.0b013e3181f45179

Weiss, T., Kreitinger, J., Wilde, H., Wiora, C., Steege, M., Dalleck, L., & Janot, J. (2010). Effect of Functional Resistance Training on Muscular Fitness Outcomes in Young Adults. *Journal of Exercise Science & Fitness, 8*(2), 113-122. doi: Doi 10.1016/S1728-869x(10)60017-2

Whitehurst, M. A., Johnson, B. L., Parker, C. M., Brown, L. E., & Ford, A. M. (2005). The benefits of a functional exercise circuit for older adults. *Journal of Strength and Conditioning Research, 19*(3), 647-651.

Whyte, L. J., Gill, J. M., & Cathcart, A. J. (2010). Effect of 2 weeks of sprint interval training on health-related outcomes in sedentary overweight/obese men. *Metabolism, 59*(10), 1421-1428. doi: 10.1016/j.metabol.2010.01.002

Williams, N. H., Hendry, M., France, B., Lewis, R., & Wilkinson, C. (2007). Effectiveness of exercise-referral schemes to promote physical activity in adults: systematic review. *Br J Gen Pract, 57*(545), 979-986. doi: 10.3399/096016407782604866

Wiszomirska, I., Krynicki, B., Kaczmarczyk, K., & Gajewski, J. (2014). The impact of functional training on postural stability and body composition in women over 60. *J Sports Med Phys Fitness*.

Wood, R. H., Reyes, R., Welsch, M. A., Favaloro-Sabatier, J., Sabatier, M., Matthew Lee, C., . . . Hooper, P. F. (2001). Concurrent cardiovascular and resistance training in healthy older adults. *Med Sci Sports Exerc, 33*(10), 1751-1758.
