# Supplementary material for: High intensity, circuit-type integrated neuromuscular training alters energy balance and reduces body mass and fat in obese women: A 10-month training-detraining randomized controlled trial
Source: PLoS One. 2018 Aug 23;13(8):e0202390. doi: 10.1371/journal.pone.0202390 (PMC6107179; doi:10.1371/journal.pone.0202390)
Supplement: S2 Protocol — (PDF) [file pone.0202390.s002.pdf]

ΠΡΩΤΟΚΟΛΛΟ ΜΕΛΕΤΗΣ

Προς: την Εσωτερική Επιτροπή Δεοντολογίας του Τμήματος Επιστήμης Φυσικής Αγωγής και Αθλητισμού, του Πανεπιστημίου Θεσσαλίας

Τίτλος: Οι επιδράσεις ενός ολοκληρωμένου νευρομυϊκού προγράμματος άσκησης στη σύσταση σώματος, το σωματικό βάρος και το ενεργειακό ισοζύγιο σε υπέρβαρες/παχύσαρκες γυναίκες καθιστικής ζωής.

Επιστημονικώς Υπεύθυνοι:
Αθανάσιος Τζιαμούρτας, Αναπληρωτής Καθηγητής, ΤΕΦΑΑ-ΠΘ
Ιωάννης Φατούρος, Αναπληρωτής Καθηγητής, ΤΕΦΑΑ-ΔΠΘ
Νικόλαος Ζουρμπάνος, Επίκουρος Καθηγητής, ΤΕΦΑΑ-ΠΘ

Σχέση με το Πρόγραμμα Σπουδών του ΤΕΦΑΑ:
Μεταπτυχιακή εργασία του Αλέξιου Μπατρακούλη φοιτητή του ΜΠΣ «Άσκηση και Υγεία», ΤΕΦΑΑ, ΠΘ».

Σκοπός της έρευνας
Ο κύριος σκοπός αυτής της έρευνας θα είναι να αξιολογήσει την αποτελεσματικότητα της κυκλικής ολοκληρωμένης νευρομυϊκής προπόνησης με ολόσωμες ασκήσεις χρησιμοποιώντας εναλλακτικούς τύπους άσκησης στο ενεργειακό ισοζύγιο παχύσαρκων γυναικών.

Σημαντικότητα της έρευνας
Οι κορυφαίες τάσεις στο χώρο της άσκησης παγκοσμίως αφορούν σε δραστηριότητες διαλειμματικής, κυκλικής και λειτουργικής προπόνησης (Thompson, 2014). Παράλληλα, τα πρόσφατα επιδημιολογικά στοιχεία και οι μελλοντικές προβλέψεις του Παγκόσμιου Οργανισμού Υγείας (WHO) σχετικά με τον επιπολασμό του υπέρβαρου ενήλικου πληθυσμού, υποδεικνύουν ότι ο ένας στους δύο στην Ευρώπη και οι δύο στους τρεις παγκοσμίως είναι και θα παραμείνουν υπέρβαροι (WHO, 2011). Όλα τα παραπάνω, ενισχύουν την ανάγκη μελέτης ενός νέου εξατομικευμένου μοντέλου άσκησης, το οποίο θα συνδυάζει αυτές τις τρεις μεθόδους (διαλειμματική, κυκλική, λειτουργική), προκειμένου να ερευνηθεί το κατά πόσο μπορούν να επιδράσουν στη σωματική απόδοση, στην ευεξία και στην υγεία υπέρβαρων υποκινητικών γυναικών.

Εισαγωγή και ανασκόπηση της βιβλιογραφίας
Η διαλειμματική, η κυκλική και η λειτουργική προπόνηση είναι τρεις από τις είκοσι πιο δημοφιλείς μεθόδους άσκησης παγκοσμίως, προωθώντας λιγότερο χρονοβόρες συνεδρίες σε σχέση με τις παραδοσιακές μεθόδους (Thompson, 2014). Σύμφωνα με τη θέση της Αμερικανικής Αθλητιατρικής Εταιρείας (ACSM), οι παραπάνω μέθοδοι συνεισφέρουν στη βελτίωση της καρδιοαναπνευστικής αντοχής, της μυοσκελετικής υγείας και ευρωστίας, καθώς και της λειτουργικής ικανότητας των φαινομενικά υγιών ενηλίκων (Garber et al., 2011). Μάλιστα οι επαγγελματίες της άσκησης φιλοδοξούν να παρέχουν στους πελάτες τους μέγιστα αποτελέσματα στον ελάχιστο δυνατό χρόνο και για το λόγο αυτό συνεχώς αναζητούν προπονητικές μεθόδους και πρωτόκολλα, τα οποία να είναι σε θέση να επιτυγχάνουν τους προπονητικούς στόχους σε λιγότερο χρόνο (Paton & Hopkins, 2005) από ότι κάποιες αντίστοιχες παραδοσιακές (Jones, Parker, & Cortes, 2011).

Πιο συγκεκριμένα, η διαλειμματική προπόνηση υψηλής έντασης (HIIT) αποτελεί μια εναλλακτική προπόνηση αντοχής, η οποία απαιτεί λιγότερο χρόνο από τη συνεχή μέθοδο σταθερής έντασης και επιφέρει φυσιολογικές προσαρμογές που βελτιώνουν τόσο την υγεία (Gibala, Little, Macdonald, & Hawley, 2012) όσο και τη φυσική κατάσταση (Gillen & Gibala, 2014) υγιών ενηλίκων και αθλητών. Πολλοί ερευνητές έχουν μελετήσει την επίδρασή της σε υγιείς υπέρβαρους/παχύσαρκους και σωματικά αδρανείς ενήλικες, βρίσκοντας θετικές προσαρμογές στη μέγιστη αερόβια ικανότητα (Lunt et al., 2014; Trilk, Singhal, Bigelman, & Cureton, 2011) στη σωματική σύσταση (Hazell, Hamilton, Olver, & Lemon, 2014), στην ινσουλινοαντίσταση (Whyte, Gill, & Cathcart, 2010), στο μεταβολικό ρυθμό ηρεμίας (Kelly, King, Goerlach, & Nimmo, 2013), στο ορμονικό προφίλ που σχετίζεται με την αύξηση του μεταβολισμού (Racil et al., 2013) και στο λιπιδαιμικό προφίλ (Miller et al., 2014).

Η σημασία της παραδοσιακής κυκλικής προπόνησης με αντιστάσεις έχει αναδειχθεί ευρέως μέσα από την επιστημονική έρευνα και ιδιαίτερη σημασία έχει δοθεί στη μελέτη της ευεργετικής της δράσης στη φυσική κατάσταση, στην ευεξία και στην υγεία του ενήλικου πληθυσμού (Marx et al., 2001; Waller, Miller, & Hannon, 2011). Επιπλέον, αυτή η μέθοδος άσκησης επιφέρει μείωση του ποσοστού σωματικού λίπους σε σωματικά αδρανείς γυναίκες μέσης ηλικίας με φυσιολογικό δείκτη μάζας σώματος (Ferreira et al., 2010) και βελτίωση της μυϊκής δύναμης, της σωματικής σύστασης, της περιφέρειας μέσης και της παραγωγής γαλακτικού οξέος σε υπέρβαρους άνδρες μέσης ηλικίας με ελάχιστη πρόσφατη σωματική δραστηριότητα (Paoli et al., 2010). Στην εφαρμογή της κυκλικής προπόνησης υψηλής έντασης σε υπέρβαρους μεσήλικες άνδρες, παρατηρήθηκε σημαντικότερη μείωση σε δείκτες υγείας, όπως η αρτηριακή πίεση και τα λιπίδια στο αίμα, σε σύγκριση με την παραδοσιακή προπόνηση αντοχής και της κυκλικής προπόνησης χαμηλής έντασης (Paoli et al., 2013). Μάλιστα, φαίνεται ότι επηρεάζει την ενεργειακή κατανάλωση ηρεμίας (REE) και μειώνει τις τιμές του αναπνευστικού πηλίκου (RR) 22 ώρες μετά τη λήξη της συνεδρίας (Paoli et al., 2012).

Ο συνδυασμός ερεθισμάτων καρδιοαναπνευστικής άσκησης και μυϊκής ενδυνάμωσης αποτελεί βασική μεθοδολογία στη συνταγογράφηση άσκησης για φυσική απόδοση και υγεία των υγιών μεσήλικων και ώριμων ενηλίκων (Kraemer, Ratamess, & French, 2002; Takeshima et al., 2004; Wood et al., 2001). Επιπλέον, η κυκλική προπόνηση με αντιστάσεις συνεισφέρει στη μείωση των παραγόντων κινδύνου για μεταβολικά νοσήματα και στεφανιοπάθεια, με σημαντικότερη τη βελτίωση του λιπιδαιμικού προφίλ και της ευλυγισίας υπέρβαρων και παχύσαρκων γυναικών, συγκριτικά με τη συνεχόμενη αερόβια άσκηση (Fett, Fett, & Marchini, 2009). Τέλος, διαφορετικά πρωτόκολλα κυκλικής προπόνησης σε σωματικά δραστήριες γυναίκες που περιλάμβαναν όχι μόνο ασκήσεις αντιστάσεων αλλά και επιπλέον αερόβια ή διαλειμματική άσκηση, σημείωσαν σημαντική οξεία επίδραση στις τιμές του γαλακτικού οξέος, της καρδιακής συχνότητας και της υποκειμενικής εκτίμησης της κόπωσης (Skidmore, Jones, Blegen, & Matthews, 2012).

Σχετικά με τη λειτουργική προπόνηση αντιστάσεων και την επίδρασή της σε σχέση με την αντίστοιχη παραδοσιακή σε υγιείς ενήλικες υπάρχει ένα σημαντικό κενό στη βιβλιογραφία, όσον αφορά ευρήματα που τεκμηριώνουν τις χρόνιες προσαρμογές στη φυσική απόδοση, στη σωματική και ψυχική υγεία. Γνωρίζουμε τη θετική επίδραση αυτού του είδους προπόνησης στους υπερήλικες σχετικά με τη λειτουργική ικανότητα (de Vreede, Samson, van Meeteren, Duursma, & Verhaar, 2005; Milton, Porcari, Foster, & Udermann, 2008; Whitehurst, Johnson, Parker, Brown, & Ford, 2005) και τη σωματική σύσταση (Wiszomirska, Krynicki, Kaczmarczyk, & Gajewski, 2014), αλλά και τη βελτίωση που επιφέρει στη μυϊκή ευρωστία και ευλυγισία νεαρών ενηλίκων (Kibele & Behm, 2009; Weiss et al., 2010). Τέλος, έχει παρατηρηθεί ότι τα προγράμματα λειτουργικής άσκησης αυξάνουν

τη συνέπεια και προσκόλληση των συμμετεχόντων στη συστηματική άσκηση (Williams, Hendry, France, Lewis, & Wilkinson, 2007). Ειδικά τα ομαδικά προγράμματα λειτουργικής προπόνησης υψηλής έντασης είναι πιο ελκυστικά από αντίστοιχα προγράμματα αεροβικής γυμναστικής με μουσική (aerobics) ή παραδοσιακής προπόνησης αντιστάσεων στους υπέρβαρους και παχύσαρκους, παρέχοντας περισσότερη απόλαυση, επιμονή, αποφασιστικότητα και μύηση στη συστηματική άσκηση (Heinrich, Patel, O'Neal, & Heinrich, 2014).

Μεθοδολογία

Δήλωση ηθικής
Οι συμμετέχοντες θα υπογράψουν το έντυπο συγκατάθεσης εφόσον ενημερωθούν για όλους τους κινδύνους, τις δυσκολίες και τα οφέλη συμμετοχής τους σε αυτήν την μελέτη. Όλες οι διαδικασίες θα είναι σύμφωνες με την αναθεωρημένη Δήλωση του Ελσίνκι το 2000 και θα εγκριθούν από την Επιτροπή Ηθικής και Δεοντολογίας του Τμήματος Φυσικής Αγωγής και Αθλητισμού του Πανεπιστημίου Θεσσαλίας.

Συμμετέχοντες και ερευνητικός σχεδιασμός
Ο κύριος σκοπός αυτής της έρευνας θα είναι να αξιολογήσει την αποτελεσματικότητα της κυκλικής ολοκληρωμένης νευρομυϊκής προπόνησης με ολόσωμες ασκήσεις χρησιμοποιώντας εναλλακτικούς τύπους άσκησης στο ενεργειακό ισοζύγιο παχύσαρκων γυναικών και όχι να συγκριθεί με άλλες προσεγγίσεις άσκησης που χρησιμοποιούνται στη διαχείριση βάρους. Θα χρησιμοποιηθεί ένας σχεδιασμός που θα βασιστεί σε μια ελεγχόμενη, τυχαιοποιημένη, τριών ομάδων επαναλαμβανόμενων μετρήσεων μελέτη και θα πραγματοποιηθεί μια προκαταρκτική ανάλυση ισχύος για την εκτίμηση του απαραίτητου αριθμού συμμετεχόντων. Εφόσον επιλεχθεί το απαραίτητο μέγεθος του δείγματος, οι συμμετέχοντες θα μοιραστούν τυχαία σε τρεις ομάδες: α) ομάδα ελέγχου με συμμετοχή μόνο στις μετρήσεις (C), β) ομάδα προπόνησης (TR) ή γ) ομάδα προπόνησης-αποπροπόνησης (TRD). Κατά τη διάρκεια των πρώτων 20 εβδομάδων, οι δύο παρεμβατικές ομάδες θα ακολουθήσουν το πρωτόκολλο της κυκλικής ολοκληρωμένης νευρομυϊκής προπόνησης (CINT). Στις εβδομάδες 21-40, η TR θα συνεχίσει το πρόγραμμα παρέμβασης και η TRD θα διακόψει την εφαρμογή του. Οι συμμετέχοντες θα προσκληθούν μέσω έντυπων φυλλαδίων στην τοπική κοινωνία, ανακοινώσεων στα δίκτυα κοινωνικής δικτύωσης και μέσω προφορικής κοινοποίησης. Αρχικά, οι συμμετέχοντες θα ακολουθήσουν μια ισοθερμιδική δίαιτα για μια περίοδο προσαρμογής 4 εβδομάδων όπου παράλληλα θα εξοικειωθούν με το πρωτόκολλο της CINT. Η δίαιτα θα αποτελείται από 55-60% υδατάνθρακες, 15-20% πρωτεΐνη, 20-25% λίπη και θα παρέχεται από διαιτολόγο-διατροφολόγο που θα αναλύσει τα ατομικά ημερολόγια διατροφής με ειδικό λογισμικό. Με την ολοκλήρωση των πρώτων 20 εβδομάδων, θα χρειαστεί αναπροσαρμογή στο θερμιδικό ισοζύγιο των εθελοντριών βάσει RMR και συνήθη φυσική δραστηριότητα.

Κριτήρια συμμετοχής στη μελέτη
Οι γυναίκες εθελόντριες που θα συμμετέχουν στη μελέτη πρέπει να πληρούν τα ακόλουθα κριτήρια εισόδου: α) να είναι σωματικά αδρανή άτομα (<7.500 βήματα ημερησίως, VO_2max <30 ml/kg/min, φυσική δραστηριότητα μέτριας-υψηλής έντασης <30 λεπτά ημερησίως βάσει επιταχυνσιόμετρου, β) να είναι υγιείς, προεμμηνοπαυσιακές γυναίκες ηλικίας 30-45 ετών, γ) να είναι υπέρβαρα ή παχύσαρκα 1[ου] βαθμού άτομα με Δείκτη Μάζα Σώματος (BMI) 25.1-34.9 kg/m^2, δ) να έχουν ιατρική άδεια για εντατική σωματική άσκηση, ε) να μην είναι

καπνίστριες για τουλάχιστον 6 μήνες πριν τη μελέτη, ζ) να μην ακολουθούν οποιαδήποτε διατροφική παρέμβαση ή να λαμβάνουν συμπληρωματικά διαιτητικά σκευάσματα ή/και φάρμακα (τουλάχιστον 6 μήνες πριν αλλά και κατά τη διάρκεια της μελέτης), η) να μην έχουν μειώσει το βάρος περισσότερο από 10% σε διάστημα μικρότερο των 6 μηνών πριν τη μελέτη, και θ) να μην έχουν συμπτώματα κατάθλιψης.

Κριτήρια αποκλεισμού συμμετοχής στη μελέτη
Οι συμμετέχοντες θα αποκλειστούν από τη μελέτη σε περίπτωση που: α) θα συμμετέχουν σε λιγότερο από το 80% των προγραμματισμένων συνεδριών και β) θα εφαρμόσουν οποιαδήποτε διατροφική παρέμβαση κατά τη διάρκεια της μελέτης.

Πρόγραμμα παρέμβασης
Το πρόγραμμα παρέμβασης CINT θα είναι ένα επιβλεπόμενο πρόγραμμα άσκησης για ολιγομελείς ομάδες (5-10 άτομα ανά συνεδρία) που θα πραγματοποιείται τρεις φορές την εβδομάδα με 48ωρη ανάπαυση μεταξύ των συνεδριών και με χρήση ασύγχρονης μουσικής για 40 εβδομάδες. Κατά τη διάρκεια των πρώτων 20 εβδομάδων, η προπόνηση θα δομηθεί σε 3 φάσεις που θα χαρακτηρίζονται από προοδευτικά αυξανόμενη επιβάρυνση. Μετά τις πρώτες 20 εβδομάδες (φάση 4), το πρωτόκολλο θα διατηρήσει την ένταση και τον όγκο της φάσης 3, αλλά η σχέση χρόνου άσκησης και χρόνου ανάπαυσης θα διαφοροποιείται ανά 2 εβδομάδες. Οι επιλεγμένες ασκήσεις θα εισάγουν θεμελιώδη κινητικά μοτίβα χρησιμοποιώντας το σωματικό βάρος και φορητά εναλλακτικά μέσα προπόνησης. Κάθε συνεδρία θα αποτελείται από 10 λεπτά προθέρμανσης (καρδιοαναπνευστική άσκηση χαμηλής έντασης, ασκήσεις ευλυγισίας και κινητικότητας), το κύριο μέρος όπου θα εφαρμόζεται το CINT και 5 λεπτά αποθεραπείας (ήπια βάδιση και διατάσεις) στο τέλος της συνεδρίας. Το κύριο μέρος θα αποτελείται από 8-12 ασκήσεις, οι οποίες θα πραγματοποιούνται σε μορφή κυκλικής προπόνησης και σταθμούς με προκαθορισμένα και χρονομετρημένα διαστήματα άσκησης και ανάπαυσης. Οι συμμετέχοντες θα πραγματοποιούν όσες περισσότερες επαναλήψεις μπορούν σε κάθε σταθμό με σωστή τεχνική και ελεγχόμενη, μέτρια ταχύτητα. Κάθε συνεδρία θα αποτελείται από εναλλασσόμενους σταθμούς με ασκήσεις καρδιοαναπνευστικές, αντιστάσεων και νευροκινητικές. Για τις ασκήσεις αντιστάσεων, οι συμμετέχοντες θα ενθαρρυνθούν να κάνουν χρήση ανεκτικών φορτίων κατά το ξεκίνημα της μελέτης και προοδευτικά θα μεταβούν σε υψηλότερα φορτία που θα τους επιτρέπουν πάντα να ολοκληρώσουν την απαιτούμενη διάρκεια σε κάθε σταθμό. Η καρδιακή συχνότητα θα καταγράφεται και θα παρακολουθείται μέσω συστήματος ομαδικής τηλεμετρίας μικρής εμβέλειας (Polar Team Solution) σε κάθε συνεδρία και η μέση τιμή καθώς και η μέγιστη τιμή της καρδιακής συχνότητας θα καταχωρούνται. Οι συμμετέχοντες θα καθοδηγηθούν ώστε διατηρήσουν μια ένταση άνω του 75% της μέγιστης καρδιακής συχνότητας. Οι τιμές της υποκειμενικής εκτίμησης της κόπωσης θα καταγραφούν για κάθε συμμετέχοντα σε κάθε συνεδρία τόσο στο τέλος του κάθε γύρου αλλά και ως μέσος όρος της συνεδρίας. Η ένταση της άσκησης θα υπολογιστεί ως μέση καρδιακή συχνότητα (ποσοστό μέγιστης καρδιακής συχνότητας που βρέθηκε κατά τη δοκιμασία της καρδιοαναπνευστικής αντοχής), ποσοστό της καρδιακής συχνότητας εφεδρείας, δείκτης κλίμακας υποκειμενικής εκτίμησης της κόπωσης, μέγιστη τιμή πρόσληψης οξυγόνου, μεταβολικά ισοδύναμα δραστηριότητας και συσσώρευση γαλακτικού οξέος στο αίμα για όλους τους συμμετέχοντες.

Συλλογή δεδομένων

Πριν την έναρξη της μελέτης και μετά από 20 και 40 εβδομάδες παρέμβασης (5 ημέρες μετά την τελευταία συνεδρία) θα πραγματοποιηθούν οι ακόλουθες ανθρωπομετρικές μετρήσεις: ύψος, βάρος, περιφέρεια μέσης, λόγος περιφέρειας μέσης/ισχίου, και ανάλυση σύστασης σώματος με απορροφησιομετρία ακτίνων Χ διπλής ενέργειας (DXA). Πριν, κατά τη διάρκεια και μετά από μία συνεδρία ανά συμμετέχοντα σε κάθε προπονητική φάση, θα πραγματοποιηθούν μεταβολικές μετρήσεις (συγκέντρωση γαλακτικού οξέος στο αίμα, κατανάλωση οξυγόνου κατά την άσκηση (VO_2) και αμέσως μετά (EPOC). Στις προαναφερθείσες χρονικές στιγμές θα αξιολογηθούν δείκτες απόδοσης της μυϊκής δύναμης (μία μέγιστη επανάληψη στη μηχανή οριζόντιων πιέσεων ποδιών) και της καρδιοαναπνευστικής αντοχής (δοκιμασία προοδευτικά αυξανόμενης επιβάρυνσης σε δαπεδοεργόμετρο). Επιπλέον, θα καταγραφεί η ημερήσια θερμιδική πρόσληψη μέσω ημερολόγιου διατροφής 7 ημερών καθώς και η συνήθης φυσική δραστηριότητα μέσω επιταχυνσιόμετρου για 7 ημέρες και κατόπιν προτροπής να διατηρηθούν οι ίδιες συνήθειες σε θέματα διατροφής όσο και υγείας. Η αξιολόγηση του βασικού μεταβολικού ρυθμού (RMR) θα πραγματοποιηθεί πρωινές ώρες (7:00-9:00) μέσω έμμεσης θερμιδομετρίας και εφόσον έχει προηγηθεί ολονύκτια νηστεία. Η 24ωρη RMR θα υπολογιστεί με την εξίσωση Weir. Το θερμιδικό κόστος και η κατανάλωση οξυγόνου της CINT θα μετρηθεί μέσω έμμεσης θερμιδομετρίας και χρήσης φορητού αναλυτή αεριών.

Βιβλιογραφία

de Vreede, P.L, Samson, M., van Meeteren, N.L.U., Duursma, S.A., & Verhaar, H.J.J. . (2005). Functional tasks exercise versus resistance strength exercise to improve daily function in older women: A randomized
controlled trial. *Journal of American Geriatric Society, 53*(1), 2-10.

Ferreira, F. C., de Medeiros, A. I., Nicioli, C., Nunes, J. E., Shiguemoto, G. E., Prestes, J., . . . Perez, S. E. (2010). Circuit resistance training in sedentary women: body composition and serum cytokine levels. *Appl Physiol Nutr Metab, 35*(2), 163-171. doi: 10.1139/H09-136

Fett, C. A., Fett, W. C., & Marchini, J. S. (2009). Circuit weight training vs jogging in metabolic risk factors of overweight/obese women. *Arq Bras Cardiol, 93*(5), 519-525.

Garber, C. E., Blissmer, B., Deschenes, M. R., Franklin, B. A., Lamonte, M. J., Lee, I. M., . . . American College of Sports, Medicine. (2011). American College of Sports Medicine position stand. Quantity and quality of exercise for developing and maintaining cardiorespiratory, musculoskeletal, and neuromotor fitness in apparently healthy adults: guidance for prescribing exercise. *Med Sci Sports Exerc, 43*(7), 1334-1359. doi: 10.1249/MSS.0b013e318213fefb

Gibala, M. J., Little, J. P., Macdonald, M. J., & Hawley, J. A. (2012). Physiological adaptations to low-volume, high-intensity interval training in health and disease. *J Physiol, 590*(Pt 5), 1077-1084. doi: 10.1113/jphysiol.2011.224725

Gillen, J. B., & Gibala, M. J. (2014). Is high-intensity interval training a time-efficient exercise strategy to improve health and fitness? *Appl Physiol Nutr Metab, 39*(3), 409-412. doi: 10.1139/apnm-2013-0187

Hazell, T. J., Hamilton, C. D., Olver, T. D., & Lemon, P. W. (2014). Running sprint interval training induces fat loss in women. *Appl Physiol Nutr Metab, 39*(8), 944-950. doi: 10.1139/apnm-2013-0503

Heinrich, K. M., Patel, P. M., O'Neal, J. L., & Heinrich, B. S. (2014). High-intensity compared to moderate-intensity training for exercise initiation, enjoyment, adherence, and intentions: an intervention study. *BMC Public Health, 14*, 789. doi: 10.1186/1471-2458-14-789

Jones, M. T., Parker, B. M., & Cortes, N. (2011). The effect of whole-body vibration training and conventional strength training on performance measures in female athletes. *J Strength Cond Res, 25*(9), 2434-2441. doi: 10.1519/JSC.0b013e31822817cf

Kelly, B., King, J. A., Goerlach, J., & Nimmo, M. A. (2013). The impact of high-intensity intermittent exercise on resting metabolic rate in healthy males. *Eur J Appl Physiol, 113*(12), 3039-3047. doi: 10.1007/s00421-013-2741-5

Kibele, A., & Behm, D. G. (2009). Seven weeks of instability and traditional resistance training effects on strength, balance and functional performance. *J Strength Cond Res, 23*(9), 2443-2450. doi: 10.1519/JSC.0b013e3181bf0489

Kraemer, W. J., Ratamess, N. A., & French, D. N. (2002). Resistance training for health and performance. *Curr Sports Med Rep, 1*(3), 165-171.

Lunt, H., Draper, N., Marshall, H. C., Logan, F. J., Hamlin, M. J., Shearman, J. P., . . . Frampton, C. M. (2014). High intensity interval training in a real world setting: a randomized controlled feasibility study in overweight inactive adults, measuring change in maximal oxygen uptake. *PLoS One, 9*(1), e83256. doi: 10.1371/journal.pone.0083256

Marx, J. O., Ratamess, N. A., Nindl, B. C., Gotshalk, L. A., Volek, J. S., Dohi, K., . . . Kraemer, W. J. (2001). Low-volume circuit versus high-volume periodized resistance training in women. *Med Sci Sports Exerc, 33*(4), 635-643.

Miller, M. B., Pearcey, G. E., Cahill, F., McCarthy, H., Stratton, S. B., Noftall, J. C., . . . Button, D. C. (2014). The effect of a short-term high-intensity circuit training program on work capacity, body composition, and blood profiles in sedentary obese men: a pilot study. *Biomed Res Int, 2014*, 191797. doi: 10.1155/2014/191797

Milton, D., Porcari, J. P., Foster, C., & Udermann, B. (2008). The effect of functional exercise training on functional frtness levels of older adults. *Gundersen Lutheran Medical Journal, 5*(1), 1-8.

Paoli, A., Moro, T., Marcolin, G., Neri, M., Bianco, A., Palma, A., & Grimaldi, K. (2012). High-Intensity Interval Resistance Training (HIRT) influences resting energy expenditure and respiratory ratio in non-dieting individuals. *J Transl Med, 10*, 237. doi: 10.1186/1479-5876-10-237

Paoli, A., Pacelli, F., Bargossi, A. M., Marcolin, G., Guzzinati, S., Neri, M., . . . Palma, A. (2010). Effects of three distinct protocols of fitness training on body composition, strength and blood lactate. *J Sports Med Phys Fitness, 50*(1), 43-51.

Paoli, A., Pacelli, Q. F., Moro, T., Marcolin, G., Neri, M., Battaglia, G., . . . Bianco, A. (2013). Effects of high-intensity circuit training, low-intensity circuit training and endurance training on blood pressure and lipoproteins in middle-aged overweight men. *Lipids Health Dis, 12*, 131. doi: 10.1186/1476-511X-12-131

Paton, C. D., & Hopkins, W. G. (2005). Combining explosive and high-resistance training improves performance in competitive cyclists. *J Strength Cond Res, 19*(4), 826-830. doi: 10.1519/R-16334.1

Racil, G., Ben Ounis, O., Hammouda, O., Kallel, A., Zouhal, H., Chamari, K., & Amri, M. (2013). Effects of high vs. moderate exercise intensity during interval training on lipids and adiponectin levels in obese young females. *Eur J Appl Physiol, 113*(10), 2531-2540. doi: 10.1007/s00421-013-2689-5

Skidmore, B. L., Jones, M. T., Blegen, M., & Matthews, T. D. (2012). Acute effects of three different circuit weight training protocols on blood lactate, heart rate, and rating of perceived exertion in recreationally active women. *J Sports Sci Med, 11*(4), 660-668.

Takeshima, N., Rogers, M. E., Islam, M. M., Yamauchi, T., Watanabe, E., & Okada, A. (2004). Effect of concurrent aerobic and resistance circuit exercise training on fitness in older adults. *Eur J Appl Physiol, 93*(1-2), 173-182. doi: 10.1007/s00421-004-1193-3

Thompson, W. (2014). Worldwide survey reveals fitness trends for 2015. *ACSMs Health Fit J, 18*(6), 8-17. doi: doi: 10.1249/FIT.0000000000000073

Trilk, J. L., Singhal, A., Bigelman, K. A., & Cureton, K. J. (2011). Effect of sprint interval training on circulatory function during exercise in sedentary, overweight/obese women. *Eur J Appl Physiol, 111*(8), 1591-1597. doi: 10.1007/s00421-010-1777-z

Waller, M., Miller, J., & Hannon, J. (2011). Resistance Circuit Training: Its Application for the Adult Population. *Strength Cond J, 33*(1), 16-22. doi: 10.1519/SSC.0b013e3181f45179

Weiss, T., Kreitinger, J., Wilde, H., Wiora, C., Steege, M., Dalleck, L., & Janot, J. (2010). Effect of Functional Resistance Training on Muscular Fitness Outcomes in Young Adults. *Journal of Exercise Science & Fitness, 8*(2), 113-122. doi: Doi 10.1016/S1728-869x(10)60017-2

Whitehurst, M. A., Johnson, B. L., Parker, C. M., Brown, L. E., & Ford, A. M. (2005). The benefits of a functional exercise circuit for older adults. *Journal of Strength and Conditioning Research, 19*(3), 647-651.

WHO. (2011). Global status report on noncommunicable diseases 2010 (pp. 33-37). Geneva (CH).

Whyte, L. J., Gill, J. M., & Cathcart, A. J. (2010). Effect of 2 weeks of sprint interval training on health-related outcomes in sedentary overweight/obese men. *Metabolism, 59*(10), 1421-1428. doi: 10.1016/j.metabol.2010.01.002

Williams, N. H., Hendry, M., France, B., Lewis, R., & Wilkinson, C. (2007). Effectiveness of exercise-referral schemes to promote physical activity in adults: systematic review. *Br J Gen Pract, 57*(545), 979-986. doi: 10.3399/096016407782604866

Wiszomirska, I., Krynicki, B., Kaczmarczyk, K., & Gajewski, J. (2014). The impact of functional training on postural stability and body composition in women over 60. *J Sports Med Phys Fitness*.

Wood, R. H., Reyes, R., Welsch, M. A., Favaloro-Sabatier, J., Sabatier, M., Matthew Lee, C., . . . Hooper, P. F. (2001). Concurrent cardiovascular and resistance training in healthy older adults. *Med Sci Sports Exerc, 33*(10), 1751-1758.
